# Real-Time, Light-Activated, and Multiplexed Monitoring of Base Excision Repair in Living Cells Using Chimeric d/l‑DNA Molecular Beacons

**DOI:** 10.1021/acssensors.5c00730

**Published:** 2025-08-04

**Authors:** Rosemarie Elloisa P. Acero, Charles E. Deckard, Jonathan T. Sczepanski

**Affiliations:** † Department of Chemistry, Texas A&M University, College Station, Texas 77843, United States; ‡ Department of Biochemistry and Biophysics, Texas A&M University, College Station, Texas 77843, United States

**Keywords:** base excision repair, APE1, fluorescent probes, photoactivatable, multiplexed assay

## Abstract

Base excision repair (BER) is a biologically and biomedically important cellular pathway responsible for repairing common DNA lesions. As a central member of the BER pathway, apurinic/apyrimidinic endonuclease 1 (APE1) is important in DNA repair and has been identified as a diagnostic and predictive biomarker for several diseases, motivating the development of analytical methods. However, the current repertoire of APE1 probes, the majority of which are derived from nucleic acids, are poorly suited for use in living cells and organisms, putting many promising biomedical applications of APE1 out of reach. Here, we exploit the bio-orthogonal properties of mirror-image l-DNA, together with a novel chimeric d/l-DNA molecular beacon architecture, to develop a highly versatile probe for intracellular BER, which we apply to the detection of APE1. The chimeric probe is simple to use, biostable, fast, and permits both real-time and light-controlled monitoring of APE1 activity in the nucleus of living cells, making it well suited for diverse intracellular applications. For example, we show that the probe can rapidly distinguish cells based on different APE1 expression levels and can monitor dynamic APE1 activity at single-cell resolution. Moreover, the generality of the probe design allowed for the development of a multiplexed assay for simultaneous imaging of APE1 and DNA glycosylase activities in living cells, which we used to reveal new insights into the efficacy of several prominent APE1 inhibitors. Overall, the chimeric d/l-DNA beacon probe presented in this work will be highly useful for researchers studying BER and provides a versatile toolkit for the development of improved BER-targeted therapies.

## Graphical abstract

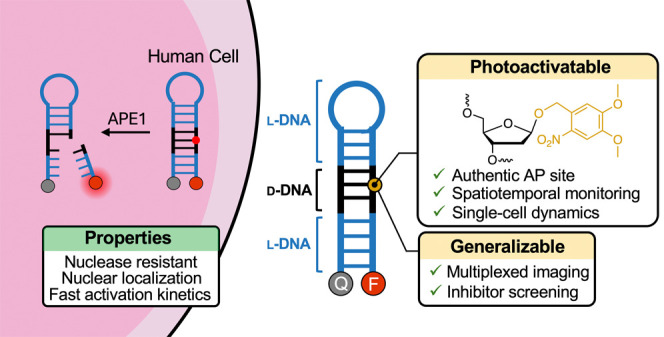

The integrity of our genomes is continuously challenged by damage arising from both exogenous and endogenous sources. If left unrepaired, DNA damage is cytotoxic and mutagenic and can lead to cancer and other diseases. The frontline defender against DNA damage is the base excision repair (BER) pathway, which is responsible for repairing most nonbulky nucleobase lesions including oxidations, alkylations and depurination, as well as misincorporated bases arising during DNA replication.^1,2^ The first step of BER is recognition and excision of a damaged base by a lesion-specific DNA glycosylase, resulting in the formation of an apurinic/apyrimidinic (AP) site (Figure 1a). AP endonuclease 1 (APE1) then cleaves the damaged strand 5′ to the AP site, allowing for incorporation of the missing nucleotide(s) by polymerase β. Finally, the remaining nick is sealed by a DNA ligase to complete the repair.^3^

**Figure 1:**
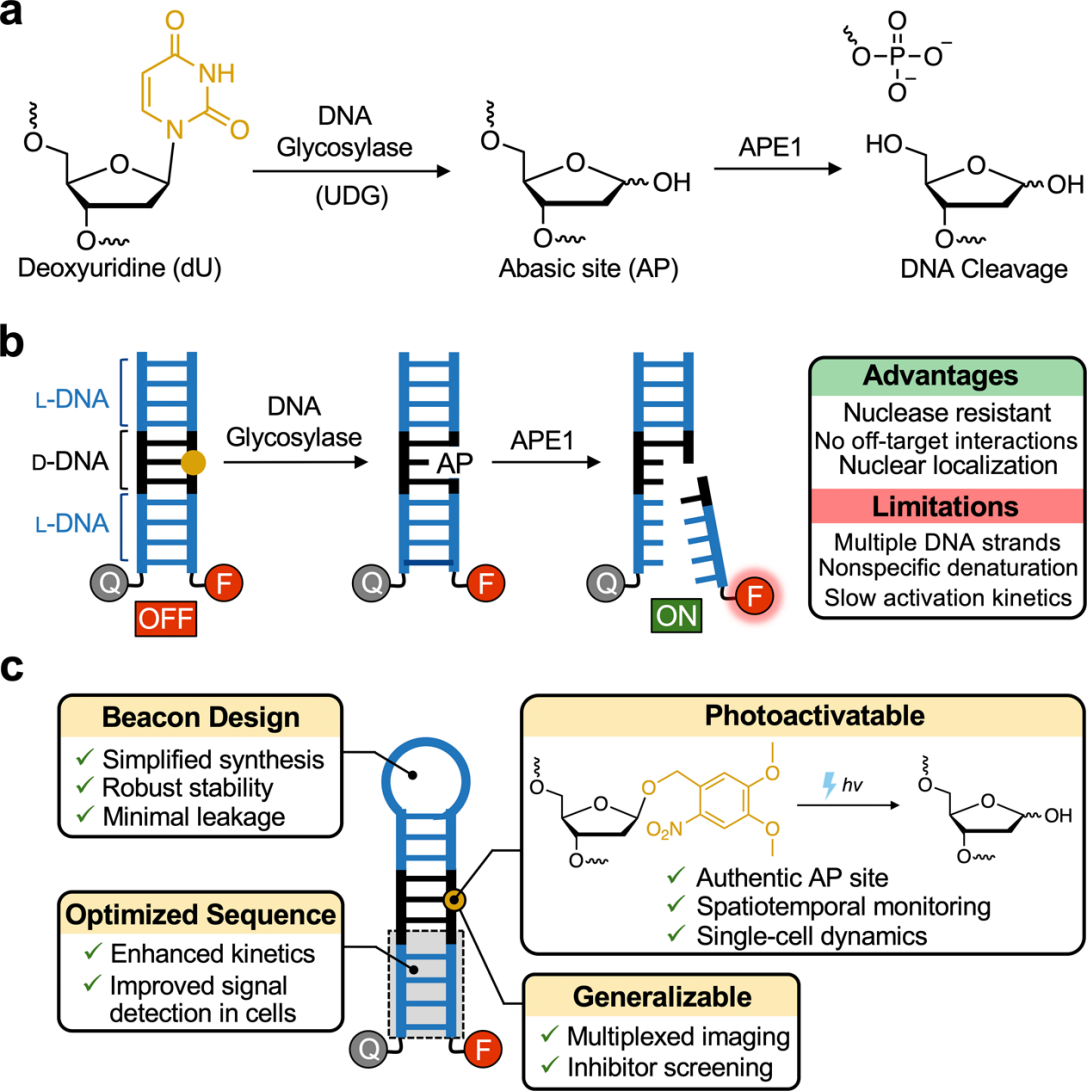
(a) Initial steps of the BER pathway. Shown here is the recognition and excision of uracil in DNA by uracil DNA glycosylase (UDG) followed by cleavage of the resulting AP site by APE1. (b) Previous chimeric d/l-DNA probes for BER were based on a duplex structure that presented several practical limitations. (c) In this study, we describe a novel chimeric d/l-DNA molecular beacon probe for BER that integrates several improvements into the previous chimeric probe design, leading to greatly enhanced intracellular performance and utility.

By processing AP sites in DNA, APE1 plays a central role in the BER pathway and, thus, is essential for maintaining genome integrity.^4^ In addition to BER, APE1 also participates in redox signaling and regulation of transcription factors and has been linked to other DNA repair pathways as well.^5–7^ A role for APE1 in RNA metabolism has also been reported.^8^ Importantly, aberrant expression or function of APE1 has been linked with various human diseases, including immunodeficiency,^9^ cellular senescence,^10^ and cancer.^6^ Indeed, APE1 is found to be overexpressed in a variety of cancers, and increased APE1 activity is correlated with resistance to a diverse range of therapies, as well as patients’ poor prognosis.^11,12^ Thus, APE1 is an attractive target for both diagnostic and therapeutic applications.^6^

The biological significance of APE1, along with its diverse roles in disease, has created a need for analytical techniques that can track APE1 activities. Notably, molecular probes that are capable of measuring APE1 activity within living cells and organisms hold great promise. Such probes can enable studies of APE1 biology and related diseases in a physiological context, allow for rapid assessment of APE1 activity between different populations of cells, and facilitate cell-based screening to identify modulators of APE1 function. However, the current repertoire of APE1 probes is poorly suited for these applications. Notably, as most probes for APE1 (and BER enzymes in general) are constructed from nucleic acid–based components, nuclease degradation and other off-target interactions remain a major barrier toward reliable intracellular operation.^13^ Moreover, delivery and retention of nucleic acid–based probes into the nucleus, where APE1 primarily resides, has proven challenging, thereby limiting detection efficiency and overall utility.^14–16^ Although some success has been achieved recently using approaches rooted in DNA nanotechnology, these methods tend to rely on complex signal generation pathways involving multiple nucleic acid and/or protein components and, in some cases, additional enzymes to transduce APE1 activity into a detectable signal.^14–19^ These complex designs require technical expertise that is beyond the average laboratory and are not easily translatable to clinical applications, such as high-throughput phenotypic screening of APE1 modulators. Indeed, the inability to screen for and to validate APE1 inhibitors in living systems represents a major roadblock toward the development of effective treatment for APE1-mediated diseases.^13,20^ Therefore, there is still an urgent need to develop APE1 probes having the simplicity, biostability, and versatility needed to support a diverse range of intracellular applications.

Our laboratory previously reported a strategy for generating intracellular probes of BER by exploiting mirror-image l-DNA, the bio-orthogonal enantiomer of natural d-DNA.^21^ As shown in Figure 1b, the probe is composed of a short segment of natural d-DNA containing the target DNA lesion, embedded within an otherwise entirely l-DNA duplex. Base excision by a lesion-specific DNA glycosylase followed by APE1-mediated strand cleavage triggers disassembly of the duplex structure, which can be monitored by fluorescence. Importantly, by being constructed primarily of l-DNA, which is both nuclease resistant and less prone to unintended interactions with native biomacromolecules, this unique chimeric d/l-DNA architecture offers many advantages compared to traditional nucleic acid–based probes, especially for intracellular applications.^22^ Nevertheless, chimeric BER probes based on this duplex design had several limitations, including the need to assemble multiple strands via hybridization, high background signals due to nonspecific duplex denaturation, and slow fluorescence activation kinetics resulting from the use of unoptimized DNA sequences. Herein, we describe a chimeric d/l-DNA molecular beacon probe for BER that integrates several improvements into the previous chimeric probe design (Figure 1c). Focusing on APE1 as the target enzyme, we show that this new probe design has improved reliability, fast fluorescence activation kinetics, and is compatible with both real-time and light-controlled monitoring of BER activity in the nucleus of living cells, making it exceptionally well suited for diverse intracellular applications. Moreover, the generality of the probe design allowed for the development of a multiplexed assay for simultaneous imaging of APE1 and DNA glycosylase activities in living cells, providing a valuable platform for the screening and validation of BER inhibitors.

## Experimental Section

Detailed materials and methods can be found in the Supporting Information.

## Results and Discussion

Our previous chimeric probe design consisted of a 7-base pair (bp) d-DNA segment flanked on both sides by 10 bps of l-DNA, with the target lesion centrally positioned within the d-DNA segment (Figure 2a). We determined that a minimum of 7 d-DNA bps was required for efficient processing of the chimeric d/l-DNA duplexes by several BER proteins. However, the central positioning of the lesion within the d-DNA region was an arbitrary design choice. Therefore, we first set out to determine how the position of an AP site within the d-DNA region impacted incision by APE1. We prepared a series of chimeric duplexes as described above bearing the chemically stable AP-site analogue tetrahydrofuran (THF) at positions 3, 4, and 5 within the d-DNA domain (AP-3, AP-4, and AP-5, respectively) (Figure 2a) and treated them with APE1. We note that the reactions were carried out in a buffer designed to mimic physiological cellular free Mg^2+^ (1.0 mM).^23^ Compared to the centrally positioned AP site (AP-4), shifting the AP site toward the 3′ direction (AP-5) had no noticeable effect on APE1-mediated incision (Figures 2b and S1). In contrast, shifting the AP site toward the 5′ direction (AP-3) nearly abolished APE1 activity, indicating that a minimum of three d-DNA bps 5′ to the lesion site is required for efficient APE1 cleavage. The inability of APE1 to incise AP-3 is likely due to a structural incompatibility with the chimeric substrate rather than the effects of sequence context, which tend to have a more subtle impact on APE1 activity.^24,25^ Interestingly, APE1 was considerably more active on chimeric substrates AP-4 and AP-5 than an all d-DNA substrate D-AP-4. We previously showed that the melting temperature of a chimeric duplex is considerably less than that of an all-d-DNA duplex of the same sequence,^21^ indicating that the discontinuous helical structure resulting from the d-DNA to l-DNA transitions has a strong destabilizing effect. Prior studies have found that APE1 activity is dependent on the thermodynamic stability of the DNA substrate.^26,27^ Thus, the increased activity of APE1 on the chimeric substrates may be explained by the destabilization of the d-DNA domain surrounding the AP site, possibly leading to reduced interactions between APE1 and the cleaved product.^27^ Future studies are required to confirm this preliminary conclusion. Although the APE1 activity is dependent on the bases adjacent and opposite to the AP site,^24,25,28^ we did not attempt further sequence optimization herein.

**Figure 2:**
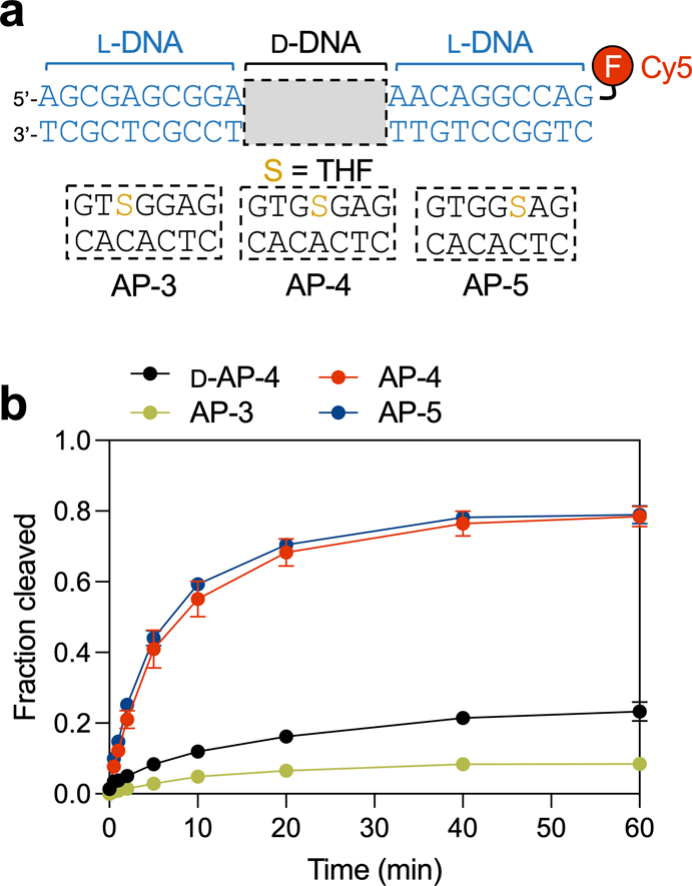
Optimization of AP site positioning. (a) Chimeric d/l-DNA duplexes with different AP site positions. See Table S1 for the complete list of oligonucleotides used in this work. d-DNA (black) and l-DNA (blue) are distinguished by color throughout the text. (b) Kinetics of APE1 (2 nM) acting on the indicated probe (200 nM) in a reaction mixture containing 20 mM Tris-acetate (pH 7.6), 50 mM KCH_3_COO, 1 mM Mg­(CH_3_COO)_2_, and 1 mM DTT. Reactions were carried out at 37 °C. Data is the mean ± standard deviation (*n* = 3). Representative gel images are shown in Figure S1.

In addition to lesion position, we considered the overall probe structure. Specifically, our prior chimeric probes consisted of two separate DNA strands that were annealed together. We realized that this design is susceptible to disassembly in the absence of BER-mediated cleavage, which could result in higher background and/or false positive signals, especially in the harsh environment of cells. Therefore, we decided to explore a molecular beacon-based design that physically links the two chimeric strands of the probe (Figure 3a). This design not only reduces the likelihood of premature disassembly of the probe, but also ensures an ideal 1-to-1 stoichiometry between the dye (Cy5) and quencher (BHQ2) moieties. Furthermore, use of a single oligonucleotide to construct the probe greatly streamlines the chemical synthesis and purification processes. We prepared a hairpin version of AP-4, referred to as AP-10, containing the same general chimeric architecture and identical dye/quencher placement (Figure 3a). APE1-mediated cleavage was found to be comparable between AP-4 and AP-10 (Figures 3b and S2), indicating that use of a hairpin structure was a feasible design strategy.

**Figure 3:**
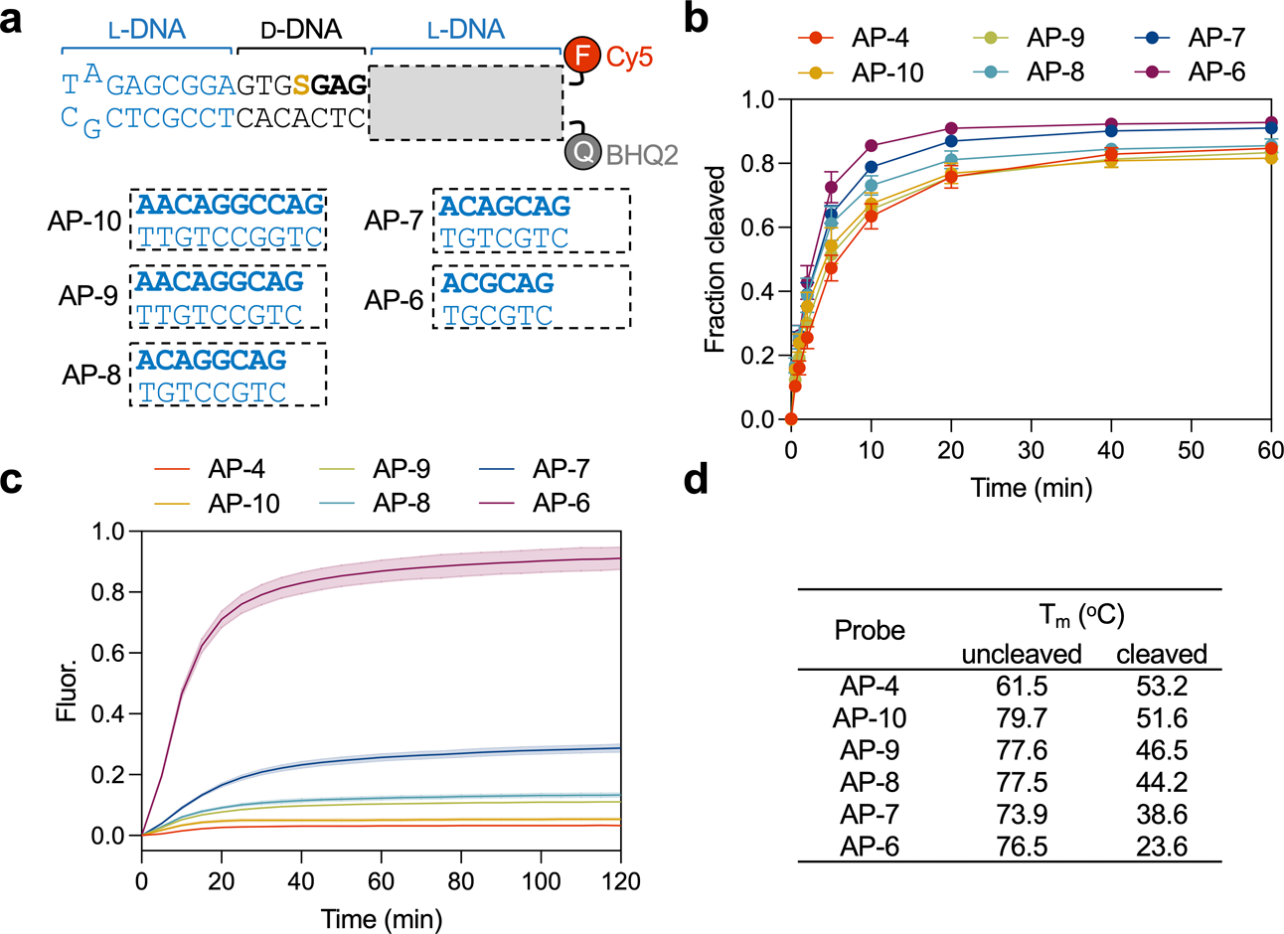
Optimization of the chimeric beacon probe design. (a) Sequence of the chimeric beacon probes tested herein. Bolded text indicates the fragment designed to dissociate upon APE1-mediated cleavage. (b) Kinetics of APE1-mediated cleavage of the indicated probe as measured by denaturing PAGE (see Figure S2). Data is the mean ± standard deviation (*n* = 3). (c) Kinetics of fluorescence activation of the indicated probe as measured by spectrofluorometry. Fluorescence (Fluor.) is reported in units such that 1.0 is the fluorescence of the probe lacking a quencher (mimicking full activation) and 0.0 is the background of the quenched probe. Shaded band shows standard deviation (*n* = 3). Reaction conditions for panel b and c are identical to those described in Figure 2b. (d) Melting temperature (*T*_m_) values of the indicated probe before and after cleavage by APE1.

We then assessed the ability of the chimeric beacon probe AP-10 to monitor APE1 activity in vitro by fluorescence, which requires both efficient APE1 incision and probe disassembly. Unfortunately, under the conditions used herein, very little fluorescent signal was observed after 2 h despite >80% of the probe being cleaved by APE1 (Figure 3c). A similar result was obtained with duplex probe AP-4 (Figure 3c). This result indicated that the thermal stability of the cleaved probe was too high to allow for efficient disassembly. Ideally, the melting temperature (*T*_m_) of the probe following cleavage by APE1 would be well below the physiological temperature of 37 °C. However, the melting temperatures (*T*_m_) of probes AP-10 and AP-4 following APE1-mediated incision were determined to be >50 °C. (Figures 3d and S3). Thus, focusing on AP-10, we synthesized a series of chimeric beacon probes with decreasing length and thermal stability within the L-DNA region 3′ to the lesion, which is designed to dissociate upon APE1-mediated cleavage (Figure 3a). Thermal denaturation studies revealed that all AP-10 variants had *T*_m_ values >70 °C prior to cleavage (Figure 3d), indicating that the truncations had little impact on the overall stability of the intact probe. Moreover, all truncated AP-10 variants were cleaved efficiently by APE1 and with comparable rates as measured by gel electrophoresis (Figures 3b and S2). Following cleavage of the truncated probes by APE1, we observed a direct correlation between the length of the incised product and its *T*_m_ (Figure 3d). Only the two shortest probes, AP-7 and AP-6, had *T*_m_ values near or below 37 °C. Consistently, only these two probes gave rise to an appreciable fluorescent signal following cleavage by APE1 (Figure 3c). Notably, cleavage of shortest probe, AP-6, resulted in a ∼10-fold increase in fluorescence, corresponding to the activation (i.e., disassembly) of >80% of the probe after 1 h (Figure 3c). This represented a ∼11-fold improvement compared to the original duplex probe AP-4. Moreover, time-dependent experiments revealed that disassembly of AP-6 (monitored by fluorescence in Figure 3c) lagged just slightly behind incision (Figure 3b), indicating that AP-6 rapidly disassembled following APE1-mediated cleavage. Thus, further truncations were not considered and we settled on AP-6 as the final design.

After confirming that AP-6 was resistant to nuclease degradation (Figure S4a), we evaluated its ability to monitor APE1 activity in living cells. HeLa cells were chosen due to their elevated APE1 expression levels and because they were used previously to evaluate chimeric probes.^14,21^ As a negative control for these experiments, we also prepared an undamaged version of the probe (T-6) by replacing the THF•A bp with a T•A bp that is not a substrate for APE1 (Figure S2). Using an unquenched version of AP-6 (AP-6_NoQ_; Table S1), we first monitored the subcellular localization of the chimeric beacon using confocal microscopy. This revealed that the probe rapidly localized into the cell nucleus following transfection, where it was retained for up to 6 h (Figure S4b). Similar behavior was observed previously for chimeric probes based on the duplex design.^21^ Interestingly, all-d-DNA versions of these duplexes were also found to localize into nucleus of HeLa cells shortly after transfection but were most exported to the cytosol after 2 h. These contrasting behaviors like reflect the ability of l-DNA to evade active transport mechanisms. Regardless of the underlying cause, nuclear localization represents a key advantage of the chimeric design. We also confirmed that the concentration of AP-6 employed throughout this study (10 pmol, 200 nM) was not toxic to cell for up to 24 h (Figure S4c). As expected, transfection of the undamaged T-6 control probe into HeLa cell resulted in a weak fluorescence signal that is consistent with the lack of APE1-mediated cleavage or other nonspecific degradation (Figure 4a). In contrast, transfection of probe AP-6 into HeLa cells yielded a bright fluorescence signal, indicating robust APE1-induced activation. Flow cytometry analysis revealed that probe AP-6 exhibited approximately six times higher fluorescence activation compared to the T-6 control (Figures 4b and S4d). Moreover, taking advantage of the biostability of l-DNA, we extracted the probes from the cells after imaging for further analysis by gel electrophoresis. As shown in Figure S4e, probe AP-6 was cleaved selectively at the THF modification, whereas the undamaged T-6 control probe showed negligible cleavage. These data are consistent with an APE1-dependent mechanism of fluorescence activation. Finally, we compared probe AP-6 to probe AP-4_Q_ (quenched version of AP-4), which represents our previous chimeric duplex design. The fluorescence signal generated by AP-6 was noticeably brighter than AP-4_Q_ in cells, corresponding to a ∼2-fold greater signal as measured by flow cytometry (Figures 4a,b and S4d). This result demonstrated the superiority of the optimized chimeric beacon design for intracellular BER imaging.

**Figure 4:**
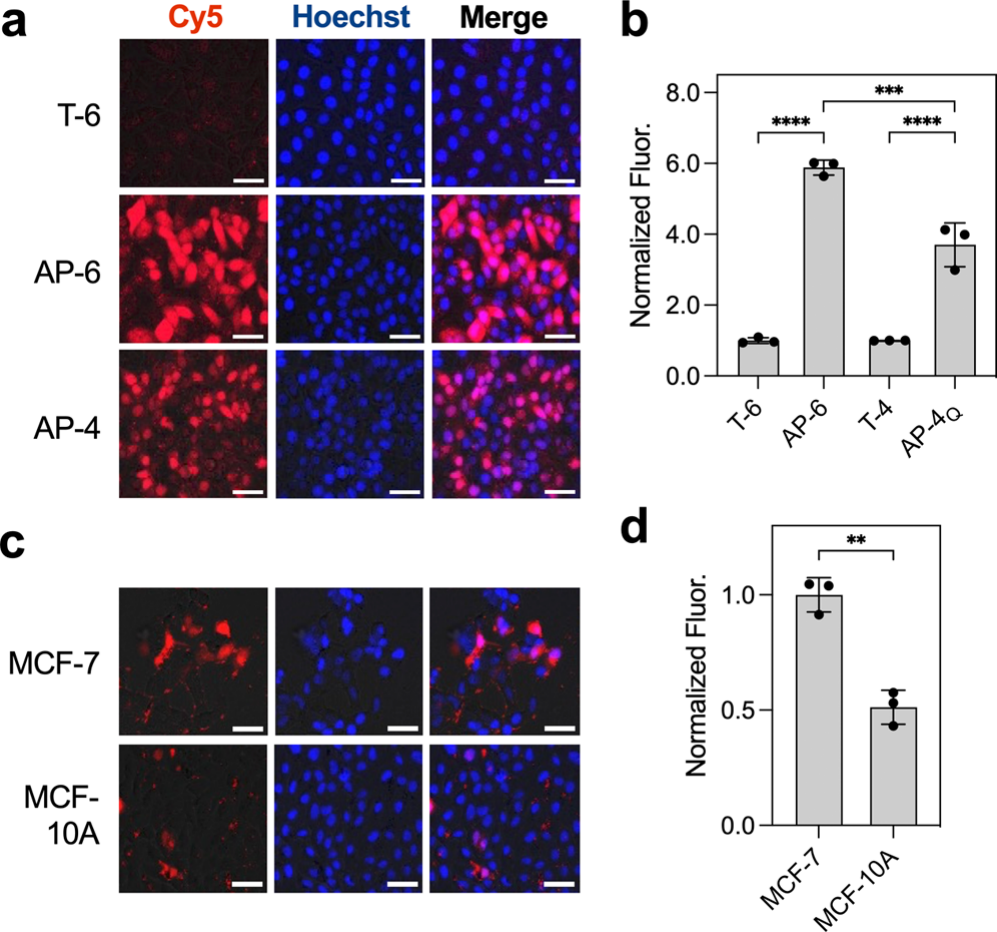
Monitoring APE1 activity in living cells using chimeric beacon probes. (a) HeLa cells were transfected with 10 pmol of the indicated probe and imaged by fluorescence microscopy 6 h later. Scale bar = 50 μm. (b) Flow cytometry analysis of cells treated under the same conditions described in panel a. Data for each probe is normalized to its respective undamaged control (T-6 or T-4). Error bar shows standard deviation (*n* = 3 biological replicates). ***P < 0.001, ****P < 0.0001. (c) MCF-7 or MCF-10A cells were transfected with 10 pmol AP-6 and imaged by fluorescence microscopy 6 h later. Scale bar = 50 μm. (d) Flow cytometry analysis of cells treated under the same conditions described in panel (c). Data is normalized to MCF-7 cells. Error bar shows standard deviation (*n* = 3 biological replicates). **P < 0.01.

We then evaluated the potential of AP-6 for monitoring APE1 in cells with different levels of APE1 expression. For this study, we monitored APE1 activity between human breast adenocarcinoma cells (MCF-7) and noncancerous human breast epithelial cells (MCF-10A). Upon transfection of chimeric beacon AP-6, a much stronger fluorescent signal was observed in MCF-7 cells relative to MCF-10A as monitored by both fluorescence microscopy and flow cytometry (Figures 4c,d and S5a–c). We note that the uptake of AP-6 was found to be similar for both cell lines using an unquenched version of the probe (AP-6_NoQ_) (Figure S5d,e and Table S1). In agreement with the fluorescence data, nuclear APE1 protein levels were determined to be ∼3-fold higher in MCF-7 cells compared to MCF-10A cells by Western blot analysis (Figure S6). These results are consistent with prior measurements of relative APE1 levels in these cell lines.^14,29^ Taken together, the above results demonstrate that AP-6 allows for the selective monitoring of intracellular APE1 activity and can distinguish between different cell lines based on their APE1 expression levels.

The biological functions of APE1 are tightly related to its subcellular localization, and the dynamics and activity of APE1 within specific subcellular compartments have been linked to clinical outcome in cancer therapy.^6^ Despite the importance of localized APE1 activity, there remains a very limited toolbox for visualizing the intracellular activity of APE1 in a spatial and temporal manner, especially within the nucleus. Therefore, we sought to develop a light-activatable version of AP-6 that would permit spatiotemporal monitoring of APE1 activity in live cells. For this, we replaced the THF modification with the photocaged AP-site precursor AP_NOV_, resulting in probe N-6 (Figure 5a). Upon exposure of N-6 to UV light (∼365 nm), the 4,5-dimethoxy-2-nitrobenzyl group (NOV) is rapidly eliminated to produce the AP-site substrate.^30^ To our knowledge, this is the first intracellular APE1 probe utilizing an authentic AP site. As shown in Figure S7, APE1 was inactive on probe N-6 in the absence of UV irradiation. However, ∼75% of the probe was cleaved by APE1 following a 5 min exposure to 365 nm UV light, indicating that the photochemical deprotection occurs rapidly. Formation of the authentic AP site was further confirmed by mass spectrometry (Figure S8). Compared to AP-6, uncaged N-6 harboring an authentic AP site showed slightly enhanced APE1-mediated cleavage kinetics as measured by denaturing PAGE (Figure S9a) and greater overall fluorescence activation in vitro (Figure S9b).

**Figure 5:**
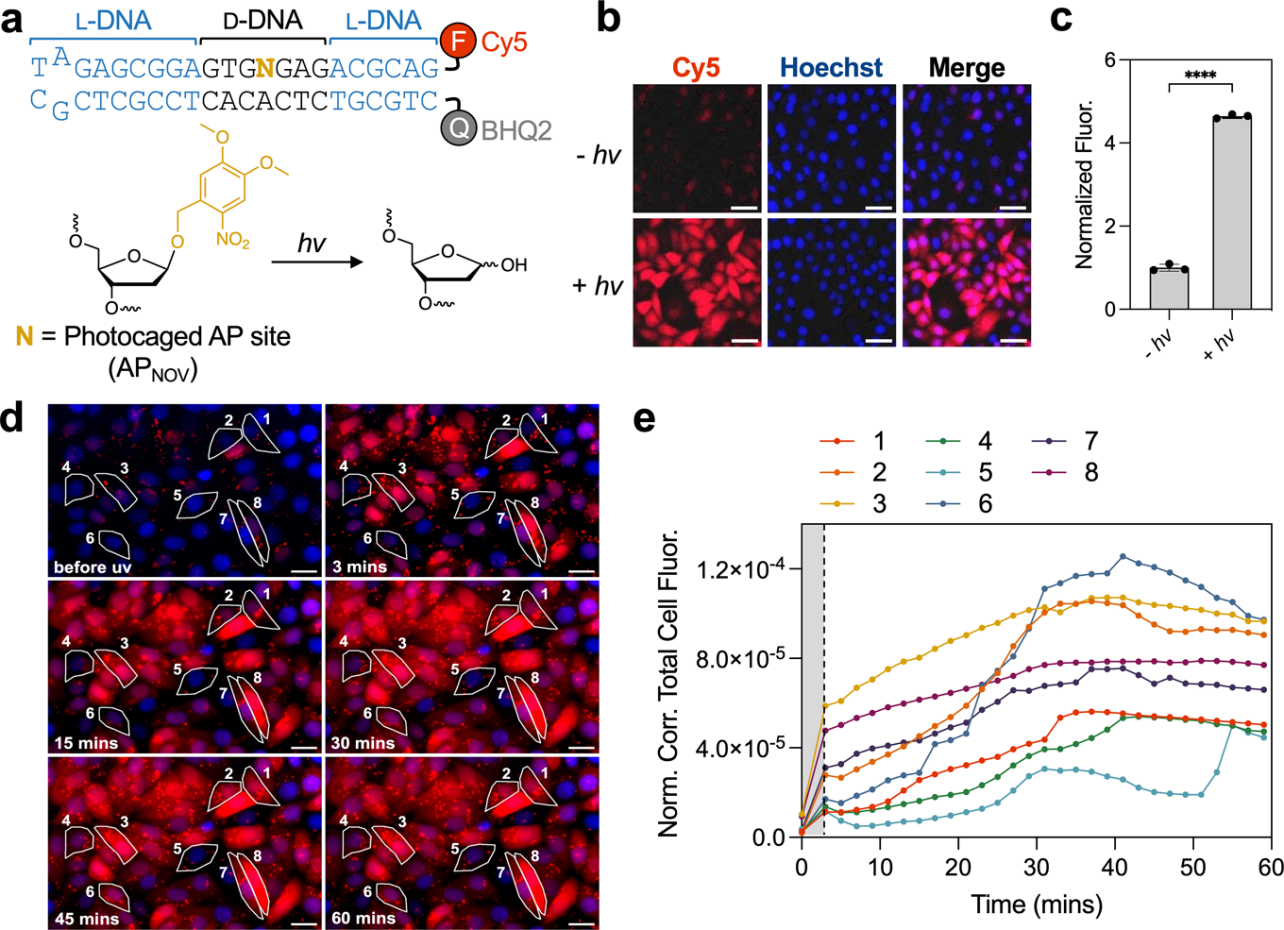
A photoactivatable probe for APE1. (a) Sequence and structure of the photocaged APE1 probe N-6. (b) HeLa cells transfected with 10 pmol N-6 and imaged by fluorescence microscopy. Cells were irradiated at ∼365 nm for 3 min (or not) and images were captured 1 h postirradiation. Scale bar = 50 μm. (c) Cells were treated under the same conditions described in panel b and analyzed by flow cytometry. Data is normalized to nonirradiated sample. Error bar shows standard deviation (*n* = 3 biological replicates). ****P < 0.0001. (d) Representative fluorescence microscopy images of HeLa cells transfected with N-6. Images were taken at the indicated time relative to UV irradiation. Scale bar = 25 μm. (e) Kinetics of N-6 fluorescence activation within selected cells in panel (d). The dashed line indicates the first measurement taken following irradiation (3 min).

We then tested probe N-6 for specific control of APE1 imaging in living HeLa cells. Cells were transfected with N-6 and, 3 h later, were irradiated with a UV light for 3 min. We note that this brief UV treatment was not toxic to the cells (Figure S10a). Irradiation of N-6 treated cells resulted in a bright fluorescence signal after just 1 h (Figure 5b). In contrast, little fluorescence was observed in cells without irradiation, indicating that the probe remained inactive until exposed to UV light. Quantification by flow cytometry showed a ∼5-fold greater fluorescence in cells treated with N-6 plus UV irradiation compared to those without irradiation (Figure 5c). As before, control probe T-6 resulted in a faint fluorescence signal that was not sensitive to UV irradiation (Figure S10b). Probe N-6 was then employed to monitor APE1 kinetics at single-cell resolution. Here, N-6 treated cells were irradiated with UV light for 3 min and imaged immediately by fluorescence microscopy, then again every 2 min over the course of 1 h (Figure 5d and Video S1 File). To account for cell-to-cell variation in transfection efficiency, N-6 was cotransfected with an unquenched version of T-6 harboring an orthogonal Cy3 fluorophore (T-6_Cy3‑NoQ_) (Figure S10c,d and Table S1). Interestingly, this analysis revealed significant heterogeneity in probe activation kinetics. As shown in Figure 5e, the fluorescence intensity of N-6 increased rapidly following UV-irradiation for a subset of the cells (e.g., cells 3 and 8), whereas the signal increased more gradually in others, with occasional “burst” periods of more rapid activity (e.g., cells 1, 2 and 6). While the exact source of this behavior is unclear and beyond the scope of this study, it may reflect cell-to-cell variability in APE1 expression levels and/or dynamic changes in other factors that influence APE1 activity and localization, such as posttranslational modifications.^31,32^ Together, these data show that UV-mediated activation of N-6 permits visualization of dynamic APE1 activity at single-cell resolution, providing a powerful tool to investigate the cellular behavior of APE1 and its responses to external stimuli.

BER assays performed in living cells hold a distinct advantage in that they report the activity of the repair machinery in its physiological context.^33,34^ In particular, multiplexed assays wherein the activity of multiple BER enzymes can be monitored simultaneously, at single cell resolution, hold great promise for the prediction and prevention of disease.^35^ Therefore, we set out to develop a simple, multiplexed assay for the simultaneous monitoring of APE1 and DNA glycosylase activity using spectrally resolved chimeric beacons. Human uracil DNA glycosylase (*h*UNG) was chosen as the target DNA glycosylase. *h*UNG is the major DNA glycosylase responsible for the recognition and excision of uracil from DNA, leading to the initiation of BER at those sites.^3,36^ To generate a chimeric beacon for *h*UNG (U-6), we simply replaced the THF modification in AP-6 with a deoxyuridine (dU) residue (Figure 6a and Table S1). Activation of U-6 first requires base excision of uracil by *h*UNG followed by APE1-mediated cleavage of the resulting AP site (Figure 1b). Functional validation of U-6 in vitro and in cells is described in the Supporting Information (see Supplementary Discussion). For multiplexing, the APE1 probe (AP-6) was labeled with Cy5 as before and the *h*UNG probe U-6 was labeled with Cy3 (Figure 6a). Cotransfection of the two probes into HeLa cells confirmed their ability to detect both APE1 and *h*UNG activity in the same cells, as seen in the fluorescence microscopy images and flow cytometry analysis (Figure 6b,c and Figure S11a). As before, minimal fluorescence activation was observed upon cotransfection of the corresponding undamaged control probes. In this assay configuration, a loss of APE1 activity will result in reduced fluorescence for both probes due to their shared requirement for APE1-mediated strand cleavage for disassembly. However, loss of *h*UNG activity will only impact the signal of U-6. Indeed, knockdown of *h*UNG by siRNA resulted in a loss of signal for U-6 without affecting APE1 probe AP-6 (Figure 6b,c), further validating probe selectivity. All fluorescence data was again validated by gel electrophoresis of the extracted probes (Figure S11b). Collectively, these results demonstrate that this multiplexed approach could be used for the simultaneous monitoring of DNA glycosylases and APE1 activities in live cells.

**Figure 6:**
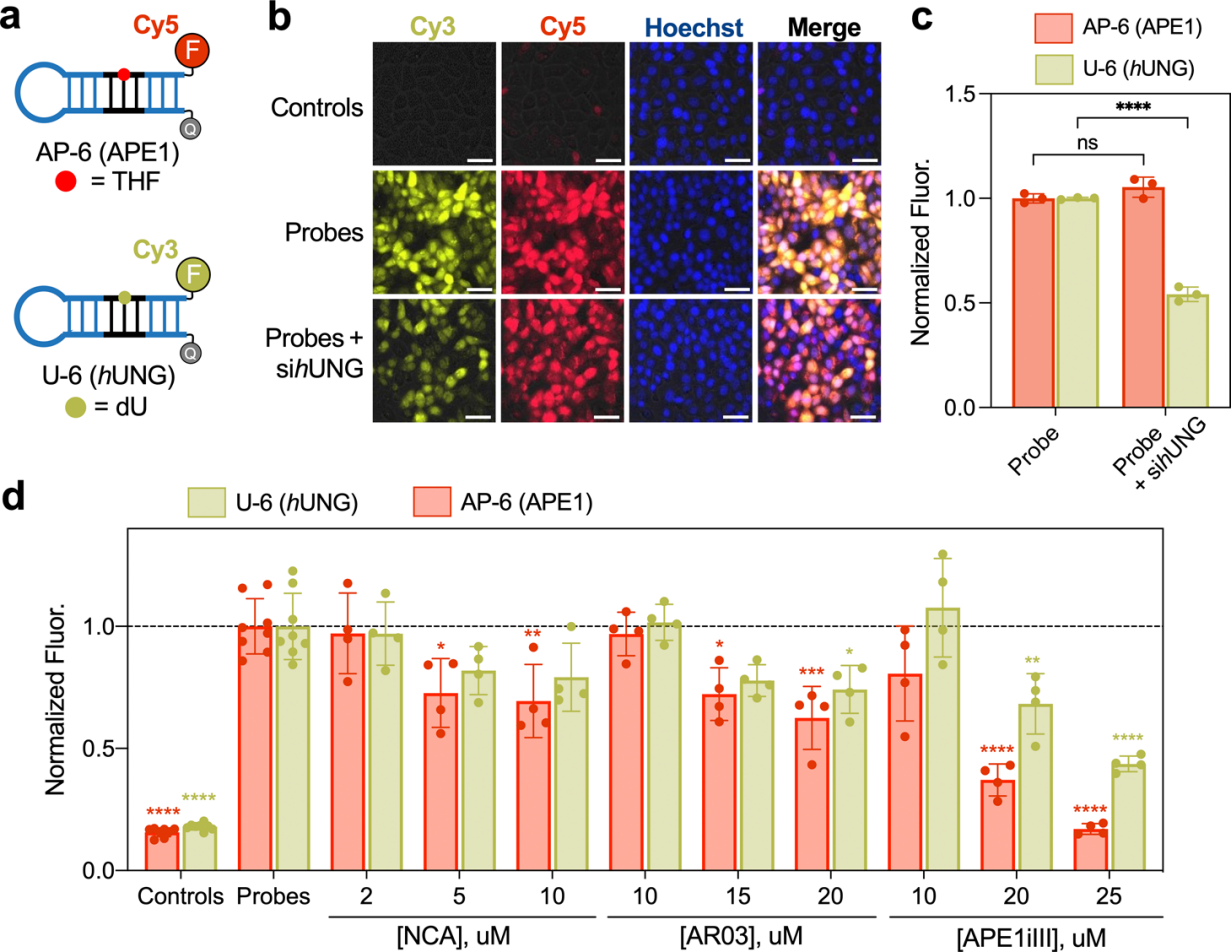
A multiplexed assay for simultaneous monitoring APE1 and *h*UNG activity. (a) APE1 and *h*UNG probes for the multiplexed assay. (b) HeLa cells were cotransfected with 10 pmol each of AP-6 and U-6 and imaged by fluorescence microscopy 6 h later. (+) si*h*UNG indicates cells that were treated with *h*UNG siRNA (both isoforms) 2 days prior to probe transfection. Scale bar = 50 μm. (c) Cells were treated under the same conditions described in panel b and analyzed by flow cytometry. Error bar shows standard deviation (*n* = 3 biological replicates). Data is normalized to samples treated with chimeric probes. (d) Evaluation of APE1 inhibitors using chimeric beacon probes. HeLa cells cotransfected with AP-6 (Cy5) and U-6 (Cy3) and analyzed by flow cytometry 6 h later. Cells were pretreated with the indicated concentration of inhibitor for 3 h prior to probe transfection. Error bars show standard deviation for cells treated with (*n* = 4) or without inhibitors (*n* = 10). Data is normalized to cells treated with chimeric probes. *P < 0.05, **P < 0.01, ***P < 0.001, ****P < 0.0001.

Finally, in order to demonstrate the utility of the chimeric hairpin probe in drug discovery applications, the multiplexed assay developed above was used to evaluate the bioactivity of APE1 inhibitors in HeLa cells. We selected 7-nitroindole-2-carboxylic acid (NCA), AR03, and APE1 inhibitor III (APE1iIII) due to their coverage in the literature, with NCA and APE1iIII being sold as APE1 inhibitors by multiple vendors (Figure S12a).^37–40^ Nevertheless, questions have been raised regarding their mode of inhibition, with recent studies suggesting that the observed in vivo activity is the result of nonspecific substrate binding and other off-target effects.^20^

We first determined IC_50_ values for these compounds in vitro using AP-6 as the functional probe for APE1 activity (Figure S12b). Our results for APE1iIII and AR03 were mostly consistent with the previously reported measurements of 2–12 μM and 2–4 μM, respectively.^20,38,39,41^ Interestingly, NCA did not inhibit APE1 under our assay conditions. Although NCA was originally reported to inhibit APE1 with an IC_50_ of ∼3 μM in vitro,^37^ subsequent studies were unable to reproduce the observed inhibition.^42^ Furthermore, recent dynamic light scattering experiments revealed that NCA forms colloidal aggregates that sequester APE1 in vitro, suggesting that prior findings of APE1 inhibition by NCA could be explained by nonspecific inhibition through compound aggregation.^20^ Given that our assay was carried out under conditions that prevent NCA aggregation, our results further support this conclusion. As expected, none of the compounds tested inhibited UDG (Figure S13).

The bioactivity of these compounds was then evaluated in HeLa cells using the multiplexed assay format. The cells were pretreated for 3 h with the indicated drug concentrations. Chimeric probes AP-6 (APE1) and U-6 (*h*UNG) were then cotransfected into the cells and their fluorescence was analyzed by flow cytometry (Figure 6d). As expected, based on our in vitro IC_50_ measurements (Figure S12), cells treated with NCA showed a very modest loss in probe signal at the highest concentrations tested, further indicating a lack of specific APE1 inhibition. Although AR03 was more effective at inhibiting APE1-mediated cleavage of AP-6 in vitro compared to APE1iIII, the opposite was true in living cells (Figure 6d), where AR03 treatment had a very limited effect. We note that attempts to increase the concentration of AR03 further resulted in significant cell death (data not shown). A recent study aimed at carefully characterizing APE1 inhibitors failed to detect interactions between AR03 and APE1 using NMR chemical shift perturbation experiments, leading the authors to conclude that AR03 inhibits APE1-mediated cleavage by binding to the DNA substrate.^20^ Indeed, 1,8-naphthyridine derivatives, such as AR03, are considered to be good DNA intercalators.^43^ Nonspecific DNA binding could explain why AR03 inhibited cleavage of the isolated AP-6 probe by APE1 in vitro but not in cells, where there exists a large excess of other nucleic acids. The high cytotoxicity of this compound is also consistent with nonspecific DNA binding (Figure S14). These results further support the conclusion that AR03 is not a selective inhibitor of APE1. Interestingly, the same NMR study referenced above also failed to detect significant interactions between APE1 and APE1iIII,^20^ again leading the authors to speculate that APE1iIII inhibits APE1-mediated cleavage through interactions with the DNA substrate. Nevertheless, in contrast to AR03, treatment with APE1iIII led to a potent, concentration-dependent loss of fluorescence for both probes (Figure 6d), indicating that the compound effectively inhibited the APE1-mediated cleavage step in cells. Thus, while the exact mode of inhibition will require further investigation, our multiplexed assay confirms that APE1iIII effectively inhibits the cleavage of AP sites in living cells. One intriguing possibility to consider is that APE1iIII binds selectively to AP sites in DNA, which would allow it to retain tight binding to the probe even in the presence of excess cellular DNA. Our observation that APE1iIII is more effective at inhibiting the cleavage of AP-6 compared to U-6 is consistent with this notion (Figure 6d). The hand-off of DNA between enzymes of the BER pathway is a highly coordinated process that prevents the exposure of mutagenic and cytotoxic repair intermediates (e.g., AP sites).^3^ Thus, the hand-off of U-6 from *h*UNG to APE1 following base excision may prevent binding of APE1iIII to the AP site leading to less inhibition of U-6 compared to AP-6. This hypothesis is supported by in vitro biochemical experiments (Figure S15). If APE1iIII is ultimately proven to bind to AP sites in DNA, our data suggests that the multiplexed assay could be used to identify such inhibitory mechanisms.

## Conclusions

In summary, we have developed a novel molecular tool for monitoring the dynamic activity of APE1 in living cells. The probe’s unique chimeric d/l-DNA design combines the simplicity of a molecular beacon with the superior biostability of l-DNA, providing a straightforward and versatile platform for intracellular imaging of APE1 activity and of BER in general. Furthermore, due to the intrinsic properties of l-DNA, the probe is easily delivered into the cell nucleus without the need for complex targeting modalities, a capability that has proven difficult to obtain with traditional nucleic acid probes of BER.^14^ We showed that these advantageous properties can be combined with controlled photoactivation to enable visualization of heterogeneous APE1 activity (i.e., kinetics) at single-cell resolution. This offers a powerful tool to study the molecular mechanisms underlying the substantial variation in DNA repair activities observed across cells,^44^ especially as it pertains to external stimuli and DNA damaging reagents. One potential limitation of the current chimeric beacon design is that it does not allow for direct monitoring of transfection efficiency, which instead must be determined indirectly using other means. In the future, the use of Förster resonance energy transfer (FRET) or other ratiometric imaging approaches would allow for the transfection efficiency of chimeric beacons to be monitored directly, thereby further improving their utility for single-cell analysis of BER activity.

Although the chimeric d/l-DNA beacon probe was designed and optimized in the context of APE1 detection, it is potentially adaptable to other BER enzymes. This was demonstrated through the construction of an intracellular *h*UNG-specific probe by simply replacing the THF modification with dU. However, we acknowledge that adapting the chimeric beacon to other DNA glycosylases may require a more extensive optimization process, as well as improved sensitivity for targets with low abundance and/or activity. We then showed that the two chimeric probes could be easily combined to generate a straightforward multiplexed assay for simultaneous monitoring of APE1 and *h*UNG activities in live cells. Given the generality of the chimeric probe design, we anticipate that this assay platform could be further expanded in the future to simultaneously monitor a variety of BER enzymes or even other DNA repair pathways. In addition to the many benefits to DNA repair research, these capabilities are expected to facilitate exciting opportunities in drug discovery, including simultaneous screening against multiple BER enzymes and more rigorous validation of mode-of-action based on multiple parameter readouts.

Finally, we used the chimeric beacon probes to evaluate the bioactivity of several reported APE1 inhibitors in live cells, revealing new insights into the efficacy of these compounds. Of the three compounds tested (NCA, AR03, and APE1iIII), only APE1iIII resulted in potent inhibition of APE1-mediated cleavage in our live-cell assay. The failure of NCA to inhibit intracellular APE1 to a significant degree is notable because this compound has been used in the past as a control to verify the selectivity of intracellular APE1 probes.^19,45,46^ However, a recent study concluded that NCA is not a true inhibitor of APE1, but instead forms colloidal aggregates that can sequester APE1, leading to nonspecific inhibition.^20^ Our findings fully support this conclusion. Thus, we urge caution regarding the use of NCA, as well as AR03, in future studies of APE1 and probes thereof. This result also highlights how screening for BER inhibitors using live-cell, high-content assays, such as the one reported herein, could eliminate nonspecific hits at an early stage of the drug discovery process, thereby increasing the reliability and efficiency of hit selection and improving the success rates in downstream clinical studies. Overall, the chimeric d/l-DNA beacon probes presented in this work will be highly useful for researchers studying APE1 biology (and the BER pathway in general) and provides a versatile toolkit for the development of improved APE1-targeted therapies.

## Supplementary Material





## Notes

Materials and methods; experimental details; supplementary figures and tables; oligonucleotide mass spectrometry data; gel electrophoresis data; supplementary discussion; video files (PDF)
Probe N-6 was monitored using fluorescence microscopy by acquiring images just before UV irradiation (3 minutes), immediately after UV irradiation, and then every 2 minutes for 1 hour. Images (31 frames) were processed and chronologically stacked using ImageJ software (v1.54g). The resulting image stack was then exported as a time-lapse video at 5 frames per second. Scale bar = 25 μm (AVI)

## §

TRACTION, The University of Texas MD Anderson Cancer Center, Houston, 77054, TX, USA
