# Supplementary material for: Real-Time, Light-Activated, and Multiplexed Monitoring of Base Excision Repair in Living Cells Using Chimeric d/l‑DNA Molecular Beacons
Source: ACS Sens. 2025 Aug 4;10(8):5655–63. doi: 10.1021/acssensors.5c00730 (PMC12379181; doi:10.1021/acssensors.5c00730)
Supplement: Supplementary file 1 [file se5c00730_si_001.pdf]

Supporting Information

Real-Time, Light-Activated, and Multiplexed Monitoring of Base Excision Repair in Living Cells Using Chimeric D/L-DNA Molecular Beacons

Rosemarie Elloisa P. Acero[1], Charles E. Deckard III[1,3], and Jonathan T. Sczepanski[1,2,*]

[1] Department of Chemistry, Texas A&M University, College Station, Texas, 77843, USA

[2] Department of Biochemistry and Biophysics, Texas A&M University, College Station, Texas, 77843, USA

[3] Current Address: TRACTION, The University of Texas MD Anderson Cancer Center, Houston, 77054, TX, USA.

S1. Supplementary Text

MATERIALS AND METHODS

General. Oligonucleotides were either purchased from Integrated DNA Technologies (Coralville, IA) or prepared by solid-phase synthesis on an Expedite 8909 DNA/RNA Synthesizer. D- and L-nucleoside phosphoramidites, CPG solid supports and all oligonucleotide synthesis reagents were purchased from Glen Research (Sterling, VA). APE1 and *E. coli* Uracil DNA glycosylase (*e*UDG) were purchased from New England Biolabs (Ipswich, MA). AR03 (Cat. # 2136) was purchased from Axon MedChem (Reston, VA). APE1 Inhibitor III (APE1iIII, Cat. # 262017) and 7-nitroindole-2-carboxylic acid (NCA, Cat. # C0496) were obtained from Sigma Aldrich (St. Louis, MO). Sulfo-Cy3 N-Hydroxysuccinimide (NHS) and Sulfo-Cy5 NHS esters were purchased from Lumiprobe (Cockeysville, MD). All other reagents were purchased from Sigma Aldrich (St. Louis, MO).

Oligonucleotide purification and labeling. All oligonucleotides used in this study are shown in Table S1. The phosphoramidite used to install the 4,5-dimethoxy-2-nitrobenzyl-caged AP site (AP_{NOV}) into probe N-6 was synthesized as previously described.[1] Black Hole Quencher 2 (BHQ2) was installed onto the 5′ ends of oligonucleotides during synthesis using the BHQ-2 phosphoramidite (Glen Research, Sterling, VA). All oligonucleotides were purified by 20% denaturing polyacrylamide gel electrophoresis (PAGE, 19:1 acrylamide:bisacrylamide). The band corresponding to the desired oligonucleotide was excised from the gel and eluted overnight at room temperature in a buffer consisting of 200 mM NaCl, 10 mM EDTA, and 10 mM Tris (pH 7.6). The solution was then filtered to remove gel fragments, and eluted oligonucleotides were concentrated using a 3 kDa pore size Amicon Ultra Centrifugal Filter unit (MilliporeSigma, Burlington, MA). Following concentration, all samples were desalted by ethanol precipitation. Sulfo-Cy5 or Sulfo-Cy3 NHS esters were conjugated to the 3′ end of PAGE-purified oligonucleotides via a 3′ amine group installed at the time of synthesis using amino modified CPG resin (3′-Amino-Modifier C7 CPG). Details of the NHS ester conjugation reaction were described in our prior work.[2] Following conjugation, samples were further purified using Agilent Infinity II HPLC system (Agilent Technologies, Santa Clara, CA) equipped with a Diode Array Detector and using a ThermoFisher DNAPac™ RP column (4 μm, 3 mm $\times$ 50 mm) maintained at 70 °C. A binary mobile phase consisting of 100 mM TEAA (Glen Research, Sterling, VA) and acetonitrile (Fisher Scientific, Pittsburgh, PA) was delivered at a flow rate of 0.6 mL/min with a total run time of 20 minutes. The gradient elution with acetonitrile (%) was as follows: 3% at 0 minutes up to 70% at

14 minutes, then 97% at 17 minutes, followed by re-equilibration to initial conditions. Final oligonucleotide concentrations were determined by absorbance at 260 nm on a NanoDrop 2000c (ThermoFisher, Waltham, MA). The identity of all novel synthetic oligonucleotides was confirmed using a Thermo Fisher Scientific Q Exactive Focus ESI mass spectrometer (Figure S16-S31). All double-stranded DNAs, including chimeric beacon probes, were annealed in folding buffer (100 mM NaCl, 20 mM Tris-HCl (pH 8.0), 1 mM DTT, and 1 mM EDTA) prior to use as previously described.[3]

Melting temperature analysis. Melting temperature experiments were conducted following methods described in our previous work.[3] APE1-cleaved probes were obtained by incubating intact probes (200 nM) with 2 nM APE1 for 1 h at 37 °C in a reaction buffer consisting of 20 mM Tris-acetate (pH 7.6), 50 mM KCH_3COO, 1 mM $Mg(CH_3COO)_2$, and 1 mM DTT. Cleaved samples were analyzed immediately without further purification.

In vitro BER assays. In vitro BER assays with APE1 were carried out in a 50 µL reaction mixture containing 200 nM of the indicated 3′ Cy5-labeled DNA substrate, 20 mM Tris-acetate (pH 7.6), 50 mM KCH_3COO, 1 mM $Mg(CH_3COO)_2$, and 1 mM DTT. Reactions were initiated by adding APE1 (2 nM final concentration) to the buffered substrates and the reaction was allowed to incubate at 37 °C for the indicated time. For BER assays with *e*UDG, a 50 µL reaction mixture containing 200 nM of the indicated 3′ Cy5-labeled DNA substrate, 20 mM Tris-HCl (pH 8.0), 0.5 mM $Mg(CH_3COO)_2$, 1 mM EDTA, and 1 mM DTT was incubated with *e*UDG (0.4 nM final concentration) at 37 °C for the indicated time. The resulting abasic site was cleaved by either heating at 90°C for 1 minute in the presence of 0.1 M NaOH or by co-incubation with 1 nM APE1. Cleaved products were resolved by 20% denaturing PAGE (19:1 acrylamide:bisacrylamide) and the gel was visualized by fluorescence emission (Cy5 excitation/emission: 635 nm/665 nm) using Typhoon FLA-9500 Multimode Molecular Imager (General Electric Co., Boston, MA). Images were quantified using ImageQuant TL software (version 8.2,Cytiva, Marlborough, MA).

APE1-mediated cleavage of the chimeric probes in solution was conducted by employing the same reaction condition described above and incubation temperature of 37 °C. Probe activation was monitored by fluorescence using a GloMax Discover multi-well plate reader (Promega Corp., Madison, WI) with excitation/emission wavelengths at 625 nm/650-670 nm for Cy5 . Fluorescence activation values (Fluor.) were corrected by adjusting the measured fluorescence signal to account for the inherent sample fluorescence and the photo-induced degradation of fluorescent signal, as described using the equation:

$$Fluor. = \frac{F_t - F_o}{F_{UQ} - F_o}$$

where Fluor. is the corrected fluorescence, F_t is the measured fluorescence at the indicated time, F_{UQ} is the measure fluorescence of an unquenched probe, and F_o is the measured fluorescence prior to addition of APE1.

For in vitro APE1 experiments involving photocaged probe N-6, the probe (200 nM) in folding buffer was initially photolyzed using a Blak-Ray B-100AP UV Lamp (UVP, Cambridge, UK) at 365 nm for the specified duration. BER assays were then carried out as previously described. In vitro BER reactions involving APE1 inhibitors (NCA, AR03, and APE1iIII) were performed using the same conditions described above with the indicated concentration of the inhibitor. Inhibitors were added from a 200× stock prepared in DMSO, giving a final DMSO concentration of 0.5% in the reaction mixture. Relative activity (RA) was calculated using the ratio of percent cleaved band in the presence ($C_{inhibitor}$) and absence (C_{DMSO}) of compound ($RA = C_{inhibitor} / C_{DMSO}$). IC_{50} values were calculated from the RA using nonlinear regression (four-parameter) by GraphPad Prism (v. 10.2.3).

Nuclease degradation assay. The indicated probe (200 nM) was incubated with either Dulbecco's Modified Eagle's Medium (DMEM; Thermo Fisher Scientific, Waltham, MA) supplemented with 10% fetal bovine serum (FBS) or 0.05 U/mL DNase I in a reaction buffer of 10 mM Tris-HCl pH 7.6, 2.5 mM $MgCl_2$, 0.5 mM $CaCl_2$ (New England Biolabs, Ipswich, MA) at 37 °C. An aliquot was obtained from each reaction at the specified time, which was quenched by the addition of EDTA to a final concentration of 10 mM and subsequent heating at 75 °C for 30 mins. Degradation products were resolved using 20% denaturing PAGE (19:1 acrylamide:bisacrylamide), and the resulting gel was imaged by fluorescence emission (Cy5 excitation/emission: 650-675 nm/675-725 nm) using Chemidoc MP Imaging System (Bio-Rad Laboratories, Hercules, CA).

Cell culture and maintenance. HeLa S3, MCF-7, and MCF-10A cells were obtained from ATCC (Manassas, VA). HeLa S3 and MCF-7 cells were cultured in Dulbecco's Modified Eagle's Medium (DMEM; Thermo Fisher Scientific, Waltham, MA) supplemented with 25 mM HEPES, 1 mM GlutaMax, and 10% fetal bovine serum (FBS; Thermo Fisher Scientific, Waltham, MA). MCF-10A cells were cultured in Mammary Epithelial Cell Growth Medium containing 0.4% BPE, 0.1% hEGF, 0.1% hydrocortisone, and 0.1% Insulin (Lonza Bioscience, Walkersville, MD). Unless otherwise

stated, cells were maintained at 37 °C in a humidified CO_2 (5%) atmosphere for the duration of the experiment.

Live-cell detection of chimeric probe activation. Cells were seeded in 96-well plates at a density of $1.2x10^4$ cells/well for HeLa cells, $2.5x10^4$ cells/well for MCF-7 cells, and $4.5x10^4$ cells/well for MCF-10A cells one day prior to transfection. Transfection solutions were prepared by adding 1 µL of 10 µM (10 pmol) probe in folding buffer and 1.25 µL of Xtremegene siRNA transfection reagent (Sigma Aldrich, St. Louis, MO) to 50 µL Opti-MEM (Thermo Fisher Scientific, Waltham, MA). In the case of multiplexed assays, 1 µL of each 10 µM probe in folding buffer was combined with 1.25 µL of Xtremegene siRNA transfection reagent. After incubating for 10 minutes at room temperature, the transfection solution was added to cells pre-washed with 2 × 100 µL PBS. Cells were maintained at 37°C in a humidified CO_2 (5%) atmosphere for 2 hours post-transfection. The culture media containing the transfection mixture was subsequently removed and each well was washed with 2 × 100 µL fresh DMEM and incubated under 100 µL DMEM for the indicated duration of the experiment.

Cell experiments with the photoactivatable probe N-6 were carried out using the same transfection protocol detailed above. However, following the 2-hour incubation with the transfection solution, the cells were washed (2 × 100 µL PBS) and subsequently incubated in 100 µL DMEM for an additional hour. The cells were then irradiated for 3 minutes using the DAPI channel (Ex: BP357/44) of EVOS FL Auto 2 Cell Imaging System (Thermo Fisher Scientific, Waltham, MA) to activate the probe. When monitoring N-6 activation kinetics, cells were co-transfected with N-6 (10 pmol) together with a mixture of $T\text{-}6_{Cy3\text{-}NoQ}$ and $T\text{-}6_{NoDye\text{-}NoQ}$ (5 pmol each; Table S1) for monitoring transfection efficiency. Probe N-6 or the control mixture ($T\text{-}6_{Cy3\text{-}NoQ}$ and $T\text{-}6_{NoDye\text{-}NoQ}$) were separately incubated with 0.75 µL of Xtremegene siRNA transfection reagent (Sigma Aldrich, St. Louis, MO) in 25 µL Opti-MEM (Thermo Fisher Scientific, Waltham, MA). After 10 minutes, the transfection solutions were mixed and added directly to the cells, which were then treated as described above. For experiments employing APE1 inhibitors, 0.5 µL of the 200× stock prepared in DMSO was added to wells containing 100 µL DMEM three hours prior to transfection (as described above).

Fluorescence microscopy images were acquired using an EVOS FL Auto 2 Cell Imaging System employing the Cy5 light cube (Ex: BP628/40; Em: BP692/40), RFP light cube for Cy3 (Ex: BP531/40; Em: BP593/40), and DAPI light cube (Ex: BP357/44; Em: BP447/60) under 20× total magnification. Prior to imaging, cells were washed with 2 × 100 µL PBS and fresh DMEM was added to each well. All images were processed using Celleste Image Analysis Software (Thermo

Fisher Scientific, Waltham, MA). For kinetic experiments employing the photoactivatable probe N-6, images were captured just before irradiation and then again every 2 mins for 1 hour after irradiation using the Cy5 channel. Probe uptake was monitored using T-$6_{Cy3\text{-}NoQ}$ by acquiring images with the Cy3 channel prior to irradiation. Images were acquired under 40× total magnification and processed using ImageJ software (v. 1.54g). Using the freehand selection and measure feature in ImageJ, the mean Cy3 and Cy5 fluorescent intensities for selected cells were obtained and used to calculate the corrected total cell fluorescence (Corr. Total Cell Fluor.). The Corr. Total Cell Fluor. for selected cells in the Cy3 channel was normalized against the maximum value observed among the selected cells to obtain a correction factor for each cell. The Cy5 Corr. Total Cell Fluor. for each time point for a given cell was then multiplied by its corresponding correction factor to give normalized Total Cell Fluor. (Norm. Corr. Total Cell Fluor.) (Figure 5e).

Flow cytometry measurements were collected using an Accuri C6 Flow Plus Cytometer (BD Biosciences, San Jose, CA). Prior to analysis, cells were rinsed twice with 100 µL PBS and detached using a 25 µL solution of 0.25% EDTA-trypsin (Thermo Fisher Scientific, Waltham, MA) at 37 °C for 5 minutes. To quench the trypsin, 25 µL DMEM supplemented with 10% FBS was added. Cy5 and Cy3 fluorescence were measured using the FL4–APC filter (Ex: 640 nm; Em: BP675/25 nm) and FL2-PE filter (Ex: 488 nm; Em: BP585/40 nm), respectively. A flow rate of 66 µL/min was used for data collection and a minimum of 10,000 events were collected for each experiment. For each sample in a given experiment, a single parameter histogram of cell count against fluorescence intensity (Cy5 or Cy3) was generated using FlowJo software (v. 10.10.0, BD Biosciences San Jose, CA). From the histogram of control samples (e.g., T-6 or T-6_{Cy3}), a gating threshold was established using the top 5% of events. Normalized fluorescence was determined by comparing the number of events above this threshold in the control and experiment samples.[4]

Probe extraction and analysis. Probes were extracted from cells and analyzed for cleavage as described in our previous work.[3] The gel was visualized by fluorescence emission (Cy5 excitation/emission: 635 nm/665 nm or Cy3 excitation/emission: 532 nm/570 nm) using a Typhoon FLA-9500 Multimode Molecular Imager (General Electric Co., Boston, MA). Images were quantified using ImageQuant TL software (version 8.2, Cytiva, Marlborough, MA).

SiRNA knockdown. For siRNA-mediated knockdown of *h*UNG, reverse transfection of *h*UNG siRNA (ID: s14678, Thermo Fisher Scientific, Waltham, MA) was performed two days prior to analyses. In a 96-well plate, *h*UNG siRNA (1 pmol) was reverse transfected into HeLa cells with a concentration of $1\text{x}10^4$ cells/well using RNAiMAX transfection reagent (Thermo Fisher Scientific,

Waltham, MA) following the manufacturer's recommended protocol. The media was replaced with fresh DMEM 24 hours later. After an additional day, the cells were either harvested for RT-qPCR analysis (as described below) or transfected with probes as described above.

RT-qPCR analysis. RT-qPCR analysis of *h*UNG knockdown or of relative APE1 expression was carried out as previously described.[3] Each reaction was performed in triplicate using sequence specific TaqMan primers for *h*UNG (Hs01037093_m1), APEX1 (Hs00172396_m1) or ACTB (Hs01060665_g1). C_T values of each sample were used to calculate ΔC_T and $\Delta\Delta C_T$, with ACTB as the internal reference. Relative gene expression levels were calculated using the $2^{-\Delta\Delta CT}$ method.

Western blotting. Nuclear proteins from 1 x 10^6 cells were extracted using NE-PER Nuclear and Cytoplasmic Extraction Reagents (Thermo Fisher Scientific, Waltham, MA) following manufacturer's protocol. Equal volumes of sample were mixed with a loading buffer containing 1x LDS sample buffer (Thermo Fisher Scientific, Waltham, MA) and 100 mM DTT and heated at 95 ^{o}C for 5 minutes. Proteins were then resolved by 10% SDS-PAGE (29:1 acrylamide:bisacrylamide) and transferred to a nitrocellulose membrane using a Trans-Blot Turbo Transfer System (Bio-Rad Laboratories, Hercules, CA). The membrane was blocked with 5% non-fat dry milk in PBST (PBS with 0.05% Tween-20) for 1 hour on a rocker at room temperature, followed by washing with 3× 5 mL PBST (3 minutes each). The membrane was then incubated overnight at 4 ^{o}C on a rocker with the primary antibody in PBST of either mouse monoclonal anti-APE1 antibody (Cat. # 13B 8E5C2, Dilution 1:1000, Thermo Fisher Scientific, Waltham, MA) or rabbit polyclonal anti-UNG antibody (Cat. # 12394-1-AP, Dilution 1:1000, Proteintech, Rosemont, IL) with mouse monoclonal anti-beta actin (Cat. # 66009-1-Ig, Dilution 1:20,000, Proteintech, Rosemont, IL) as reference. After washing with 3 × 5 mL PBST (3 minutes each), the membrane was incubated with secondary antibody in PBST of Cy3 anti-mouse IgG (Cat. # A10521, Dilution 1:10,000, Invitrogen, Waltham, MA) or Cy3 anti-rabbit IgG (Cat. # A10520, Dilution 1:10,000, Invitrogen, Waltham, MA) for 1 hour at room temperature on a rocker. Upon washing with 3 × 5 mL PBST (5 minutes each), the membrane was imaged by fluorescence emission (Cy3 excitation/emission: 520-545 nm/577-613 nm) using Chemidoc MP Imaging System (Bio-Rad Laboratories, Hercules, CA).

Nuclear localization studies. HeLa cells were seeded at 30,000 cells/well on Ibidi 8-well chamber slides (ibidi GmbH, Gräfelfing, Germany) one day prior to transfection. Unquenched AP-

6 (AP-6_{NoQ}; 10 pmol) was transfected into the cells following the protocol described above. After the indicated time, cells were washed with 5 × 150 µL PBS and fixed with freshly prepared 4% formaldehyde in PBS (150 µL) for 30 minutes. Cells were then treated with 1 µL 0.5 mg/mL Hoechst 33342 for 5 minutes, washed with 3 × 150 µL PBS, and finally soaked in 150 µL PBS. Confocal images of fixed cells were obtained using a Leica SP8 confocal microscope using a HC PL APO 40x/1.10 W motCORR CS2 water immersion objective in conjunction with a 405 nm CW laser and a 470nm–670 nm white pulsed laser. Images were processed using ImageJ software (v. 1.54g).

Cellular uptake analysis. Probes were transfected into cells following the protocol described above. Cellular uptake was analyzed as previously described by flow cytometry using an Accuri C6 Flow Plus Cytometer using the FL4–APC filter (Ex: 640 nm; Em: BP675/25 nm).

Cell viability assays. HeLa cells were plated and transfected with the indicated probe as described above, then incubated under DMEM for the specified duration. Cell viability was assessed using the Cell Counting Kit-8 reagent (CCK-8; Abcam, Cambridge, MA) following the manufacturer's protocol After the indicated duration, cells were incubated with CCK-8 solution in DMEM at 37°C for 1 hour, after which the absorbance at 450 nm was measured on a GloMax Discover multi-well plate reader (Promega Corp., Madison, WI). To correct for background signal, the absorbance of a control well containing only the CCK-8 reagent in DMEM was subtracted from each sample.

Statistical analysis. Statistical analysis was carried out on GraphPad Prism (v. 10.2.3) and presented as means and standard deviations. Datasets for a given experiment were compared using either an unpaired t-test or one-way analysis of variance (ANOVA), applying Welch's correction or Tukey's multiple comparisons test (α threshold = 0.05) to assess significant differences, respectively.

S2. Supplementary Figures

Figure S1

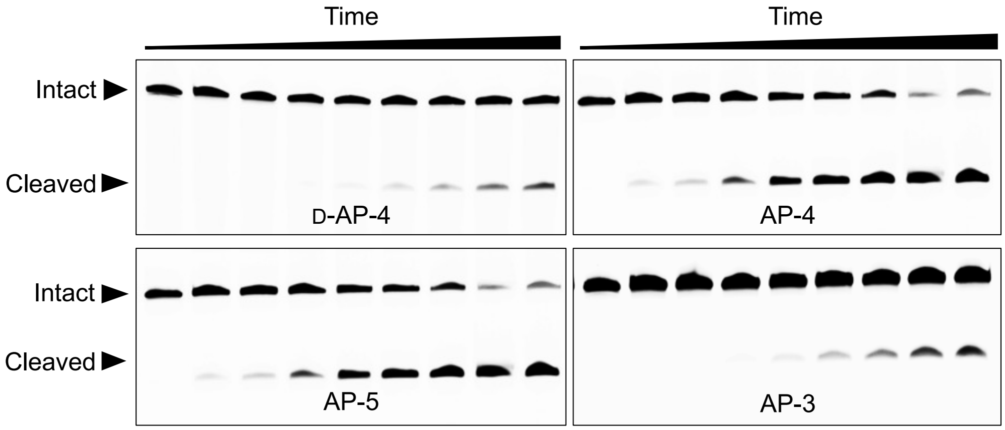

Figure S1. Kinetics of APE1-mediated cleavage of the indicated probe as measured by denaturing PAGE (20%, 19:1 acrylamide:bisacrylamide). Representative gels are shown. The indicated probe (200 nM) was treated with APE1 (2 nM) in a reaction buffer containing 20 mM Tris-acetate (pH 7.6), 50 mM KCH_3COO, 1 mM $Mg(CH_3COO)_2$, and 1 mM DTT and were carried out at 37 °C.

Figure S2.

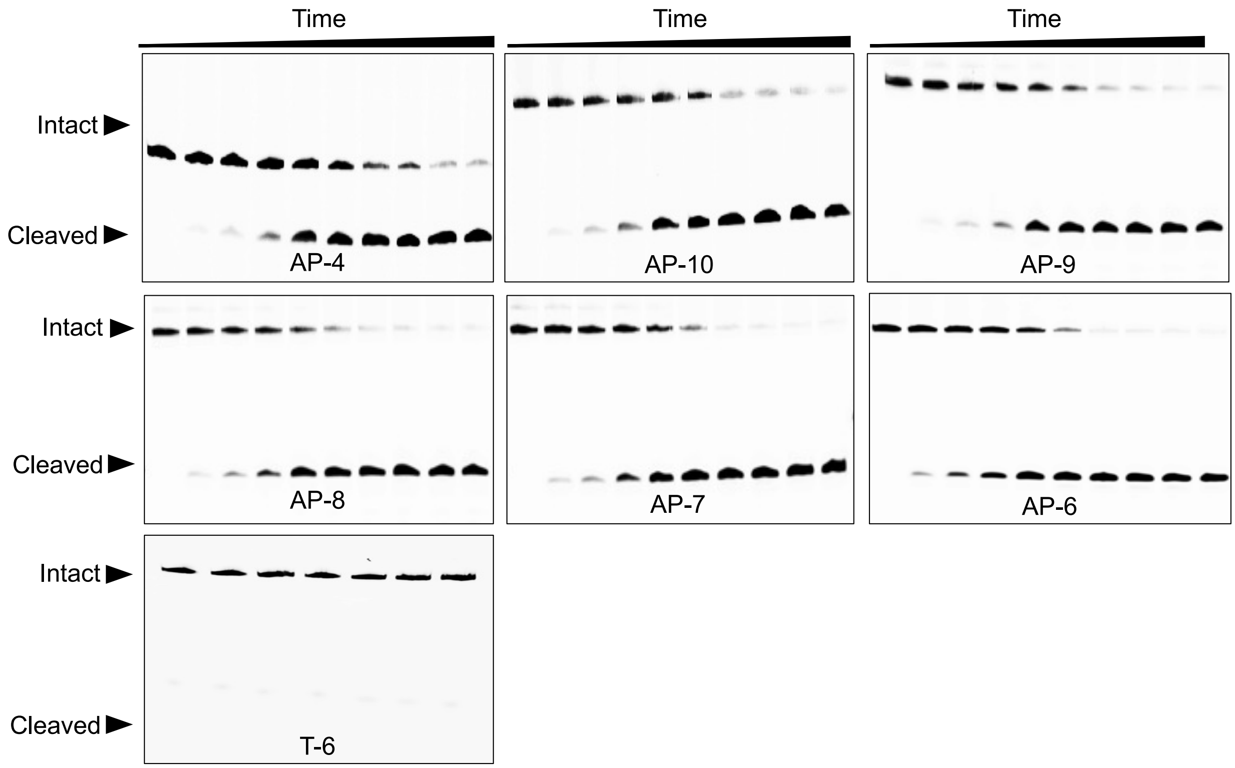

Figure S2. Kinetics of APE1-mediated cleavage of the indicated probe as measured by denaturing PAGE (20%, 19:1 acrylamide:bisacrylamide). Representative gels are shown. The indicated probe (200 nM) was treated with APE1 (2 nM) in a reaction buffer containing 20 mM Tris-acetate (pH 7.6), 50 mM KCH_3COO, 1 mM $Mg(CH_3COO)_2$, and 1 mM DTT and were carried out at 37 °C.

Figure S3

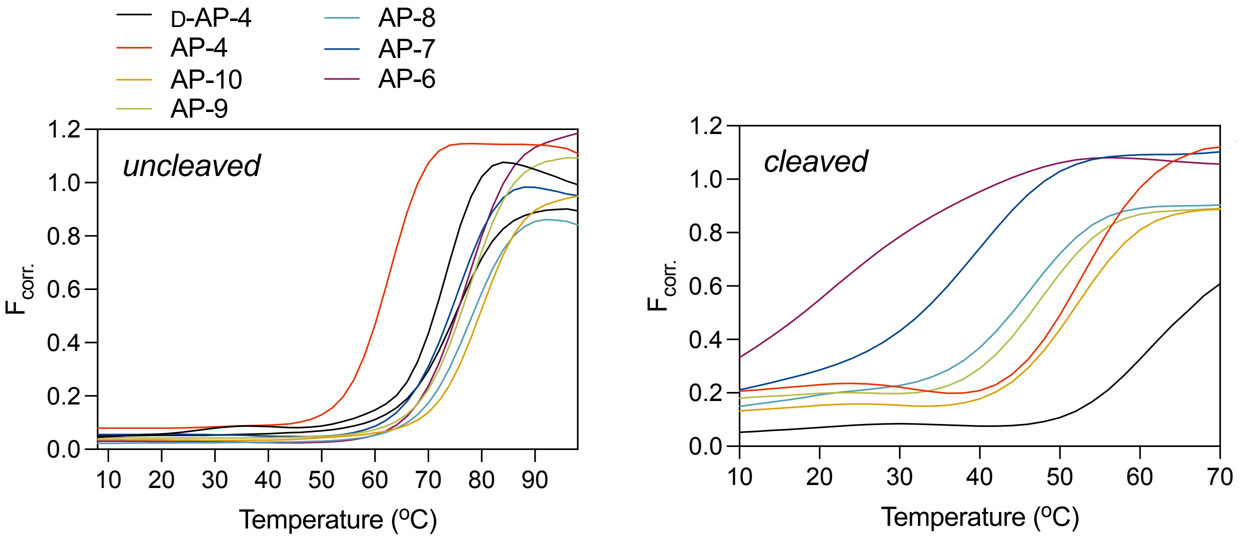

Figure S3. Fluorescence melting curves for the indicated intact (left) or cleaved (right) probe (200 nM) in a buffer containing containing 20 mM Tris-acetate (pH 7.6), 50 mM KCH_3COO, 1 mM $Mg(CH_3COO)_2$, and 1 mM DTT. Fluorescence values were corrected (F_{corr}) for background fluorescence and temperature-dependent effects as previously described.[3] Melting temperature (T_m) values reported in Figure 3d were determined by plotting the second derivatives of the fluorescence data in excel. The intersection of the curve on the x-axis was considered the T_m.

Figure S4

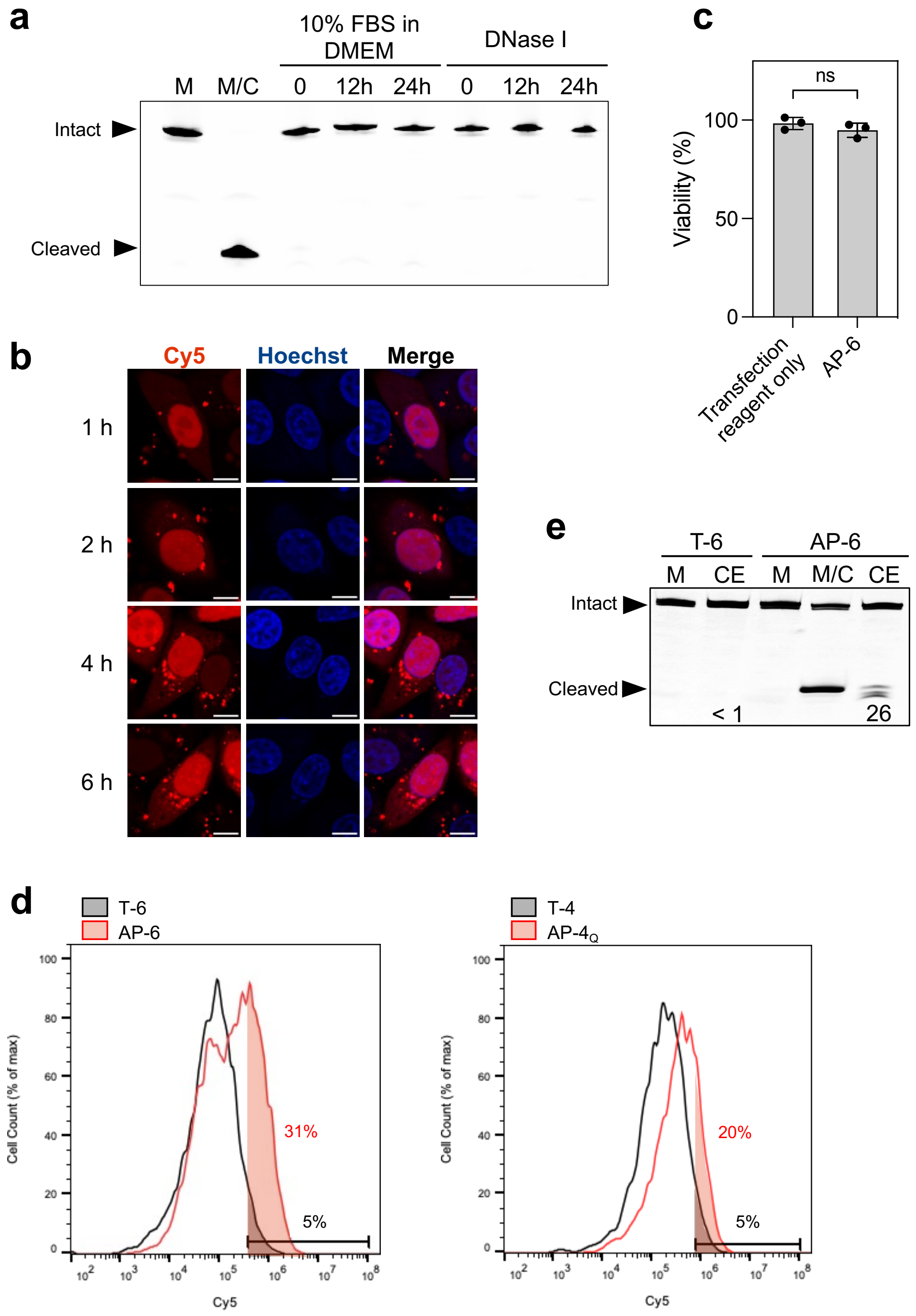

Figure S4. (a) Denaturing PAGE analysis of AP-6 in different biological environments. The indicated probe (200 nM) was incubated either in DMEM supplemented with 10% FBS or with 0.05 U/mL DNaseI in a reaction buffer containing 10 mM Tris-HCl (pH 7.6), 2.5 mM $MgCl_2$, and 0.5 mM $CaCl_2$ at 37 °C for indicated period of time. M = intact marker; M/C = cleaved marker. (b) Representative fluorescence confocal microscopy images of HeLa cells that were transfected with

200 nM of the unquenched chimeric hairpin probe AP-6_{NoQ} for the indicated time. Scale bar is 10 μm. (c) Viability of HeLa cells 24 hours post-transfection with probe AP-6 as measured by CCK-8 assay. Signals were normalized to untreated HeLa cells. Data is mean ± standard deviation (n = 3 biological replicates). (d) Flow cytometry histogram of HeLa cells treated with the indicated probe. A gating threshold was established using the top 5% of events for the controls (T-6 or T-4). Normalized fluorescence in Figure 4b was determined by comparing the number of events above this threshold in the experiment samples (AP-6 and $AP4_Q$). (e) Denaturing PAGE analysis of probes extracted from HeLa cells. M = marker for the intact probe; M/C = marker for the cleaved probe; CE = cell extract. Values below CE lanes indicate the percent of cleaved probe.

Figure S5

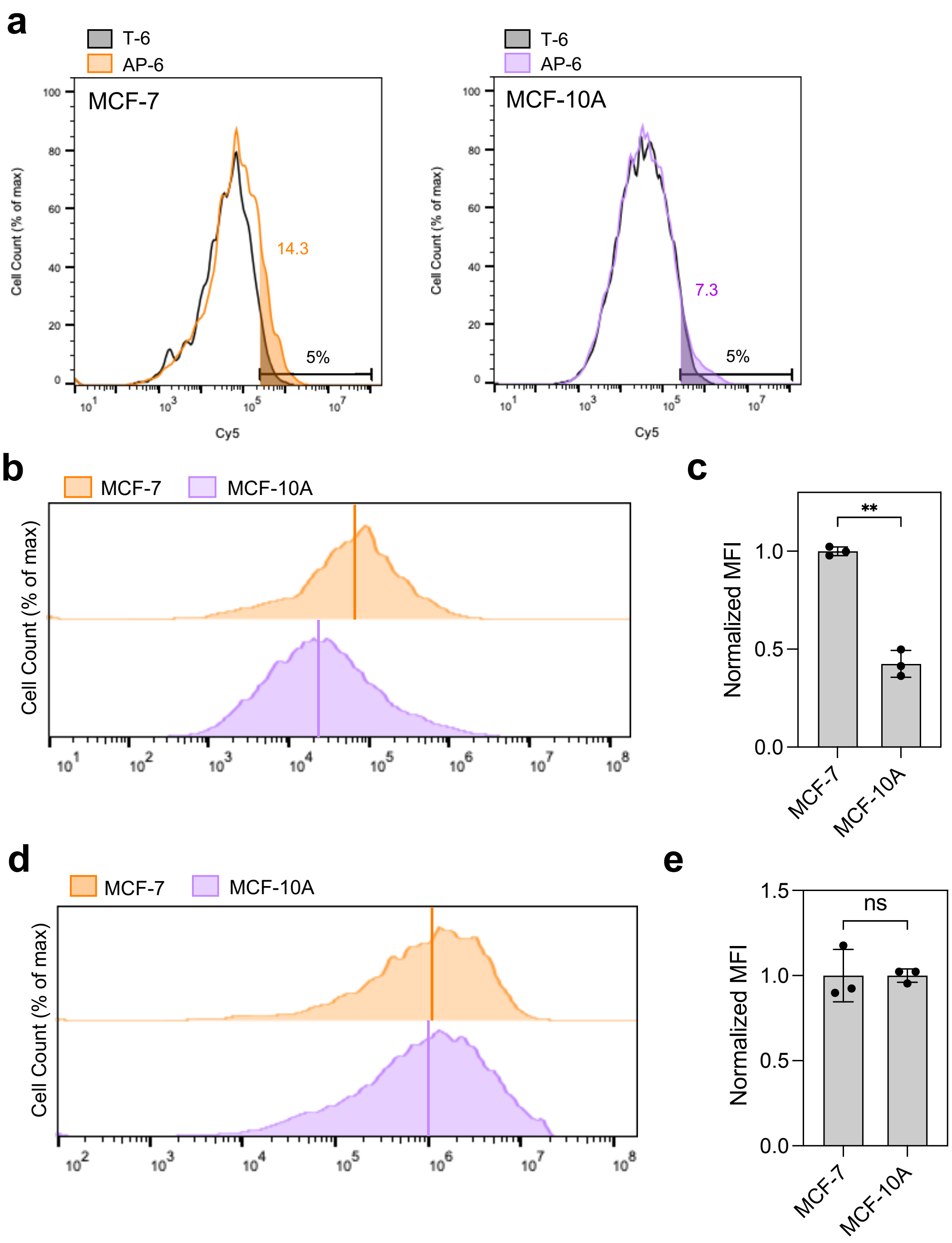

Figure S5. (a) Flow cytometry histograms of either MCF-7 or MCF-10A cells treated with the indicated probe. A gating threshold was established using the top 5% of events for the control (T-6). Normalized fluorescence in Figure 4d was determined by comparing the number of events above this threshold in the experiment samples (AP-6 and AP4$_Q$). (b) Flow cytometry histogram of probe AP-6 following transfection into either MCF-7 or MCF-10A cells. The vertical line indicates

the median value. (c) Quantification of the flow cytometric data in (b). Data (median fluorescence intensity, MFI) is normalized to MCF-7 cells and is presented as mean ± standard deviation (n = 3 biological replicates). $^{**}P < 0.01$. (d) Flow cytometry histogram of probe AP-6_{NoQ} following transfection into either MCF-7 or MCF-10A cells. The vertical line indicates the median value. (e) Quantification of the flow cytometric data in (d). Data (median fluorescence intensity, MFI) is normalized to MCF-7 cells and is presented as mean ± standard deviation (n = 3 biological replicates).

Figure S6

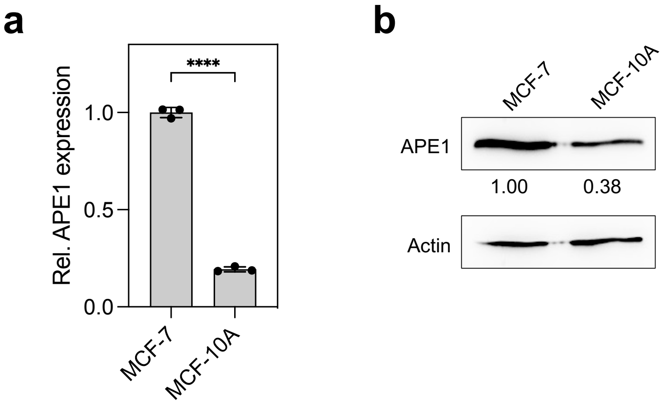

Figure S6. (a) Relative expression of APE1 in MCF-7 and MCF-10A cells as measured by RT-qPCR. Relative APE1 expression was calculated using the $2^{-\Delta\Delta Ct}$ method and normalized to MCF-7 cells. Data is mean ± standard deviation (n = 3 biological replicates). *****P* < 0.0001. (b) Western blot analysis of APE1 protein levels in MCF-7 and MCF-10A cells. Values below each lane indicate the relative APE1 protein levels (normalized to actin). Uncropped gel images are presented in Figure S32a.

Figure S7

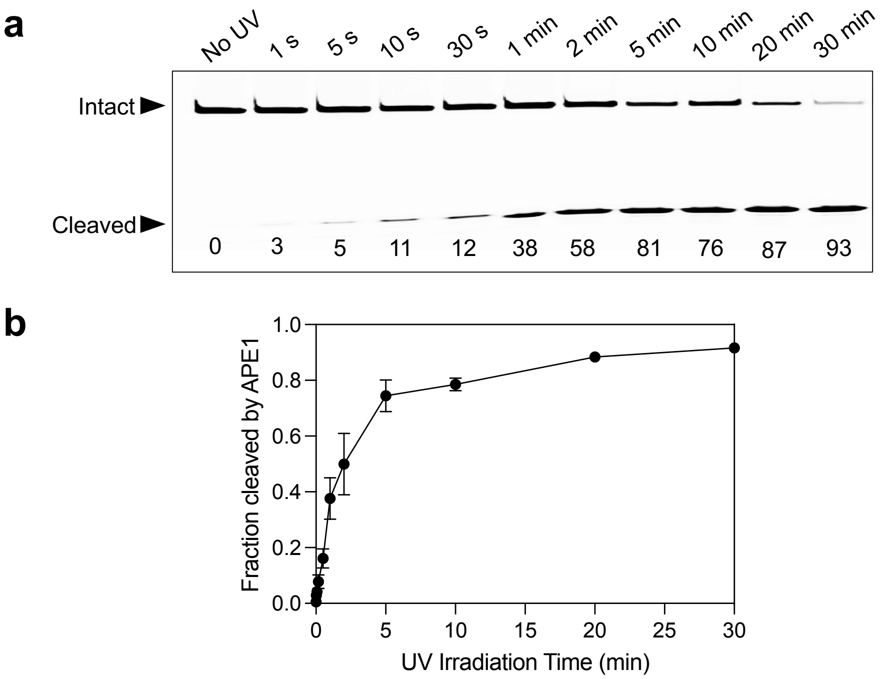

Figure S7. (a) Denaturing PAGE (20%, 19:1 acrylamide:bisacrylamide) analysis of probe N-6 after being irradiated (365 nm) for the indicated time and subsequently treated with APE1. APE1 cleavage reactions were carried out for 30 minutes under the conditions described in the caption for Figure S1. The fraction of N-6 cleaved by APE1 is indicated under each lane. (b) Plotting of the data represented in panel a. Error bars show standard deviation (n = 3).

Figure S8

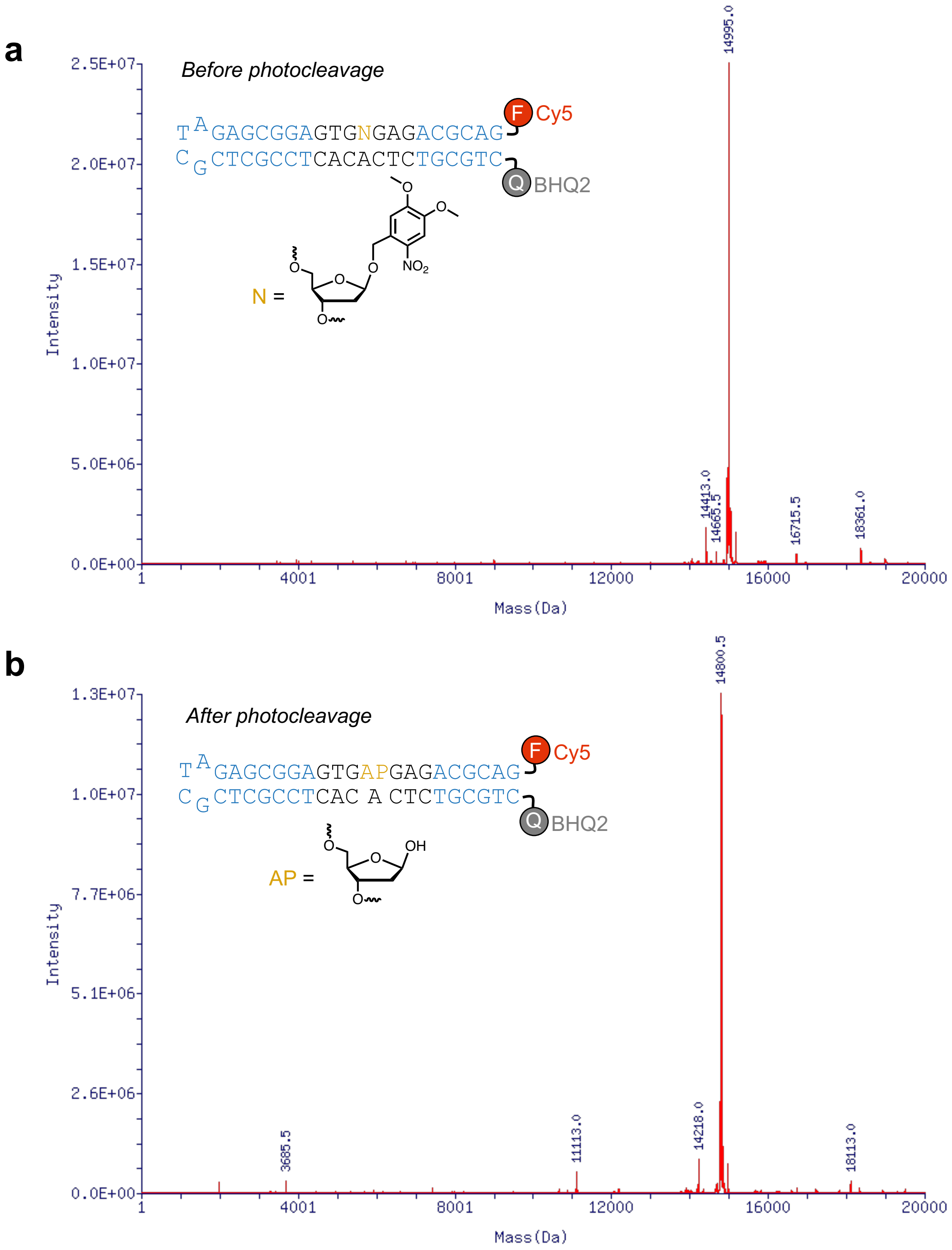

Figure S8. (a) ESI-MS of probe N-6 prior to UV treatment. Mass calculated: 14994.5; Mass found: 14995.0 Da. (b) ESI-MS of probe N-6 following a 30-minute exposure to UV light (365 nm). Mass calculated: 14799.8 Da; Mass found: 14800.5 Da.

Figure S9

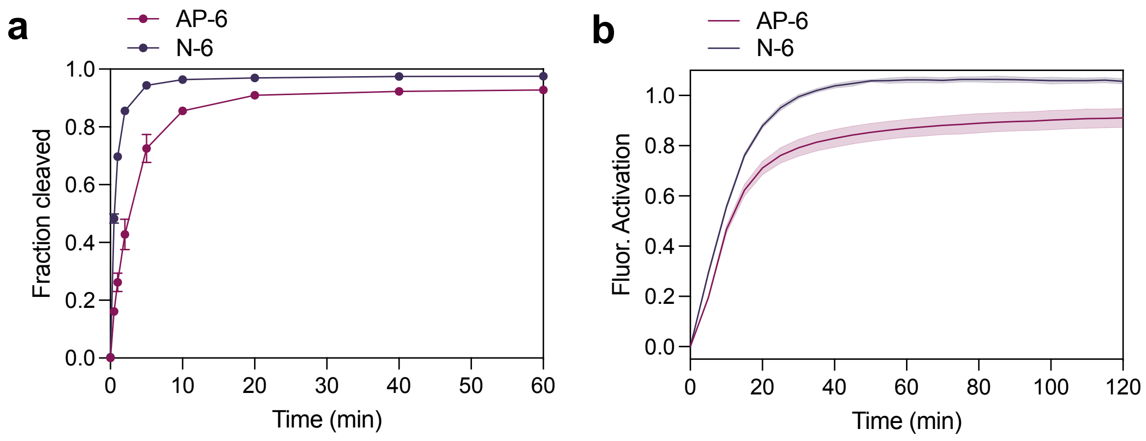

Figure S9. (a) Kinetics of 2 nM APE1 acting on 200 nM of the indicated probe in a reaction buffer containing 20 mM Tris-acetate (pH 7.6), 50 mM KCH_3COO, 1 mM $Mg(CH_3COO)_2$, and 1 mM DTT at 37 °C. N-6 was photolyzed (365 nm) for 30 minutes prior to incubation with APE1. Error bar shows standard deviation (n = 3). (b) Kinetics of fluorescence activation of the indicated probe as measured by spectrofluorometry. Shaded band shows standard deviation (n = 3).

Figure S10

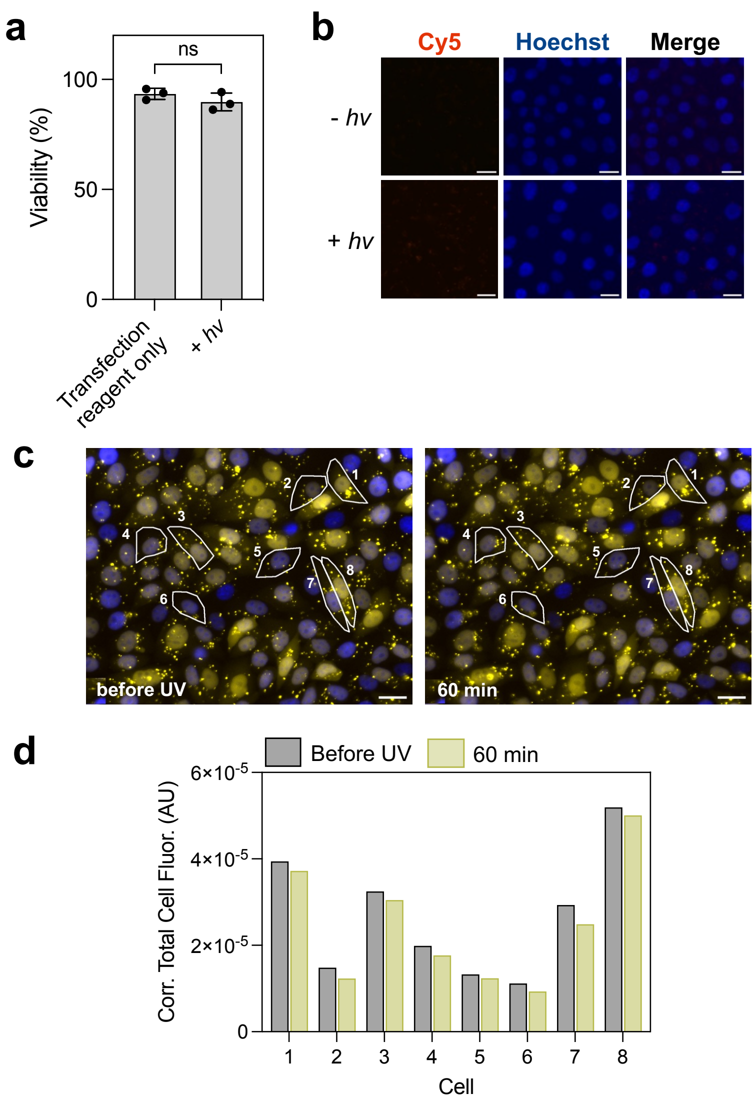

Figure S10. (a) Viability of HeLa cells transfected with probe N-6 as measured by CCK-8 assay. Cells were transfected with 200 mM N-6 for 2 hours before replacing the media with fresh DMEM. The CCK-8 assay was performed 1-hour post-irradiation. Signals were normalized to untreated HeLa cells. Data is mean ± standard deviation (n = 3 biological replicates). (b) Representative fluorescence microscopy images of HeLa cells transfected with control probe T-6. Cells were irradiated at ~365 nm for 3 minutes (or not) and images were captured 1 hour later. Scale bar is 25 µm. (c) For the experiment depicted in Figure 5d, N-6 was co-transfected with T-6$_{Cy3\text{-}NoQ}$ and the Cy3 channel was imaged just before UV treatment and again after 60 minutes. The Corr. Total Cell Fluor. obtained from these images (d) was used to correct the Cy5 signal (i.e., N-6) for the corresponding cell as described in the Materials and Methods section. The corrected data (Norm. Corr. Total Cell Fluor.) is presented in Figure 5e.

Figure S11

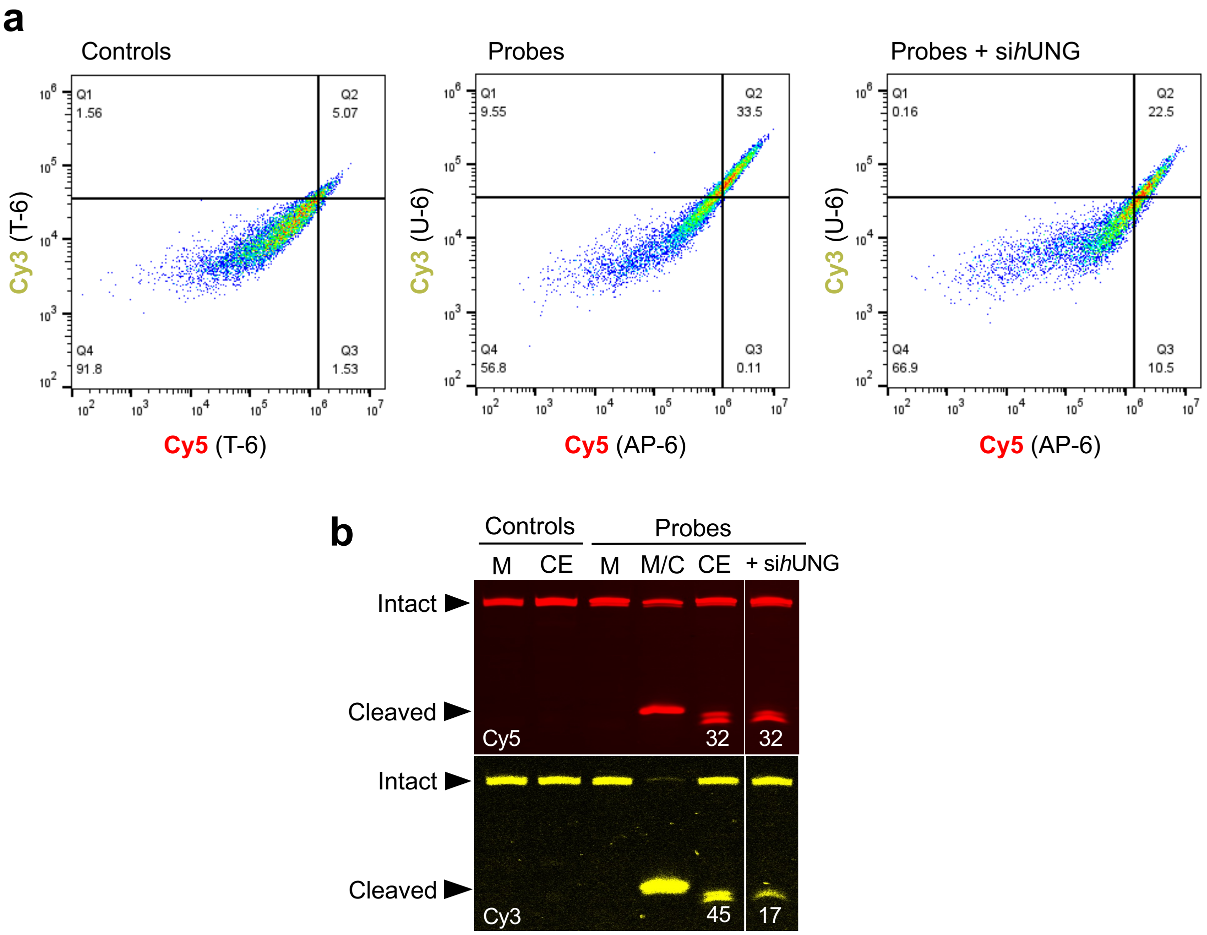

Figure S11. (a) Representative flow cytometry data for the co-treatment of HeLa cells with probe AP-6 (Cy5) and U-6 (Cy3) (or associated controls) under the indicated conditions. (b) Denaturing PAGE analysis of AP-6 (top) and U-6 (bottom) extracted from HeLa cells. M = marker for the intact probe; M/C = marker for the cleaved probe; CE = cell extract; +si*h*UNG = extract from *h*UNG siRNA treated cells. Below each lane is the percent of cleaved probe.

Figure S12

a

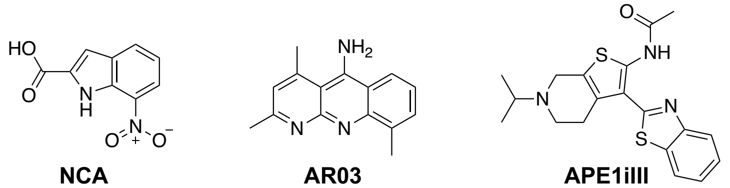

NCA No APE1 activity

AR03 IC_{50} = 14.21 ± 1.93 μM

APE1iIII IC_{50} = 20.56 ± 0.86 μM

b

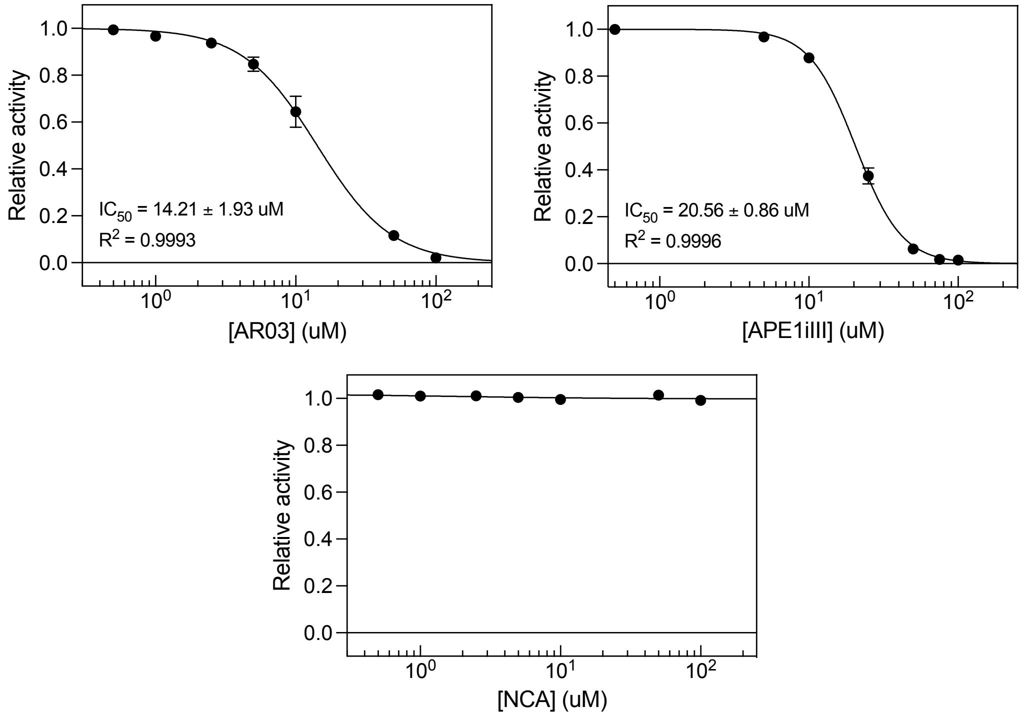

Figure S12. (a) Structures of the APE1 inhibitors used in this work. IC_{50} values were determined independently using AP-6 (panel b). (b) Dose response curves for the inhibition of APE1 by inhibitors AR03, APE1iIII, and NCA. Reactions were performed using the same conditions described in Figure S1 with the indicated concentration of the inhibitor. Inhibitors were added from a 200× stock prepared in DMSO, giving a final DMSO concentration of 0.5% in the reaction mixture. Error bars show standard deviation (n = 3 biological replicates).

Figure S13

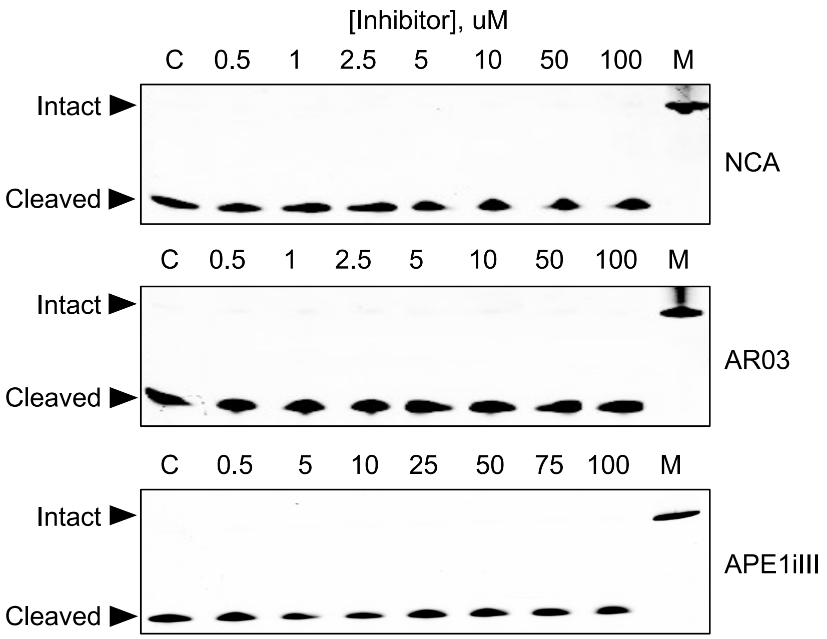

Figure S13. Denaturing PAGE (20%, 19:1 acrylamide:bisacrylamide) analysis of probe U-6 following treatment with *e*UDG in the presence of APE1 inhibitors. Reactions were performed using the same conditions described in Figure S1 with the indicated concentration of the inhibitor. The AP sites were cleaved by heating at 90 °C in the presence of 0.1 M NaOH prior to gel loading. Inhibitors were added from a 200× stock prepared in DMSO, giving a final DMSO concentration of 0.5% in the reaction mixture. C = DMSO control only; M = intact probe marker.

Figure S14

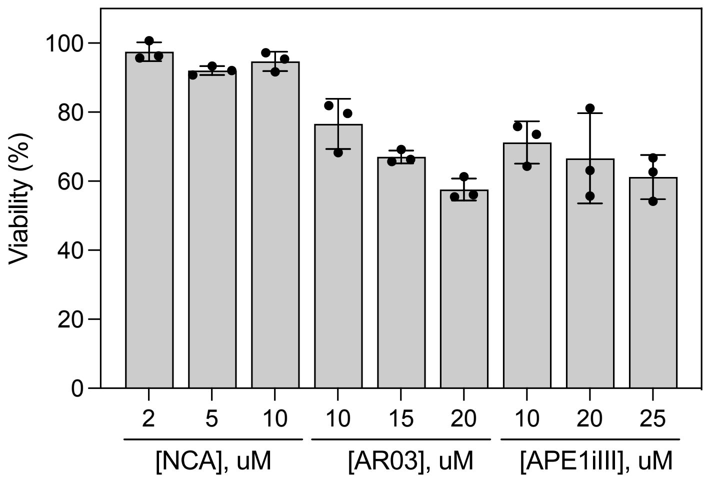

Figure S14. Viability of HeLa cells treated with the indicated concentration of inhibitor as measured by CCK-8 assay. The CCK-8 assay was carried out 9 hours post-treatment. Signals were normalized to untreated HeLa cells. Error bars show standard deviation (n = 3 biological replicates).

Figure S15

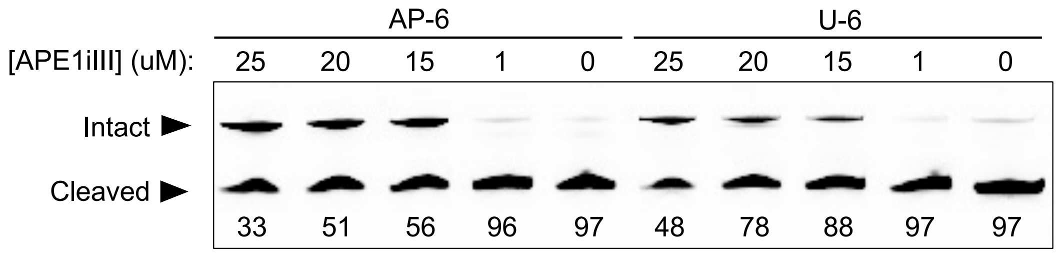

Figure S15. Denaturing PAGE (20%, 19:1 acrylamide:bisacrylamide) analysis of probes AP-6 and U-6 following treatment with *e*UDG/APE1 in the presence of increasing concentrations of APE1iIII. Reaction mixtures contained 200 nM of the indicated probe, 10 nM *e*UDG and 1 nM APE1 in a buffer consisting of 20 mM Tris-acetate (pH 7.6), 50 mM KCH_3COO, 1 mM $Mg(CH_3COO)_2$, and 1 mM DTT. The amount (%) of cleaved product is indicated under each lane.

Figure S16

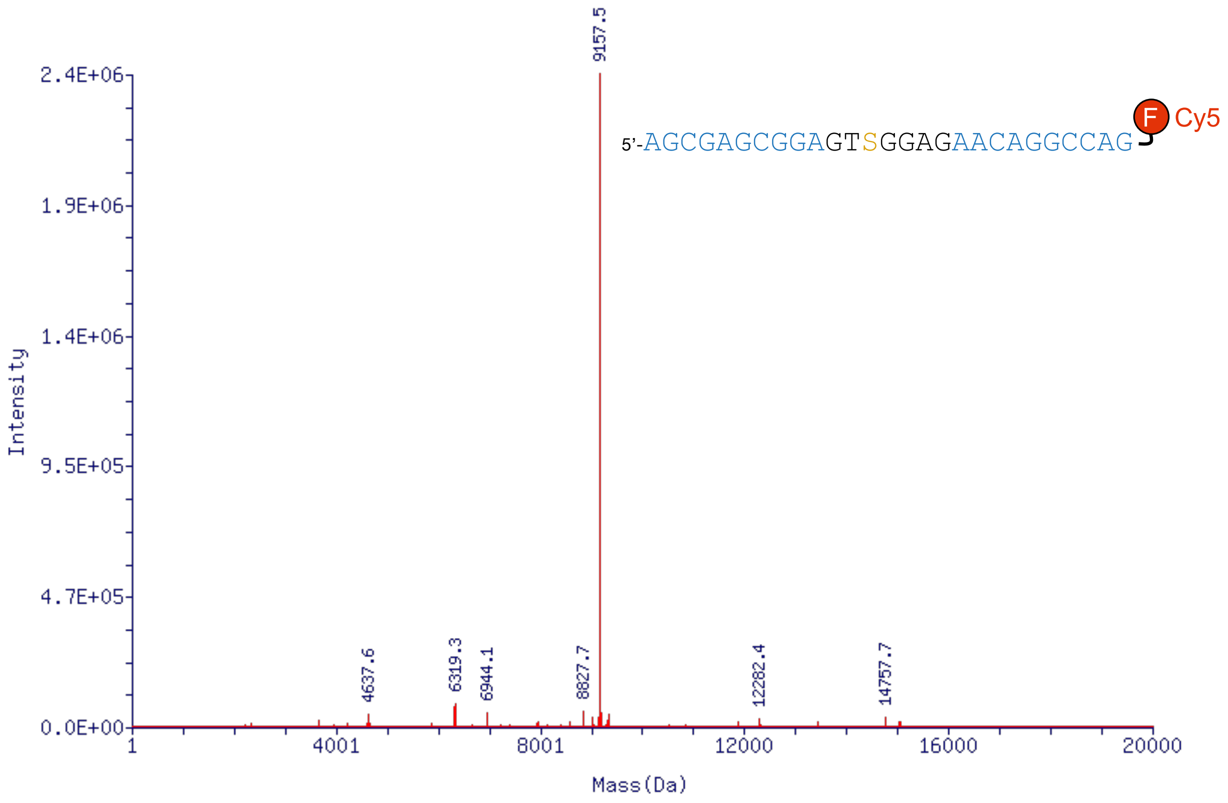

Figure S16. ESI-MS of AP-3 (top strand). S = THF modification. Mass calculated: 9157.8 Da; Mass found: 9157.5 Da.

Figure S17

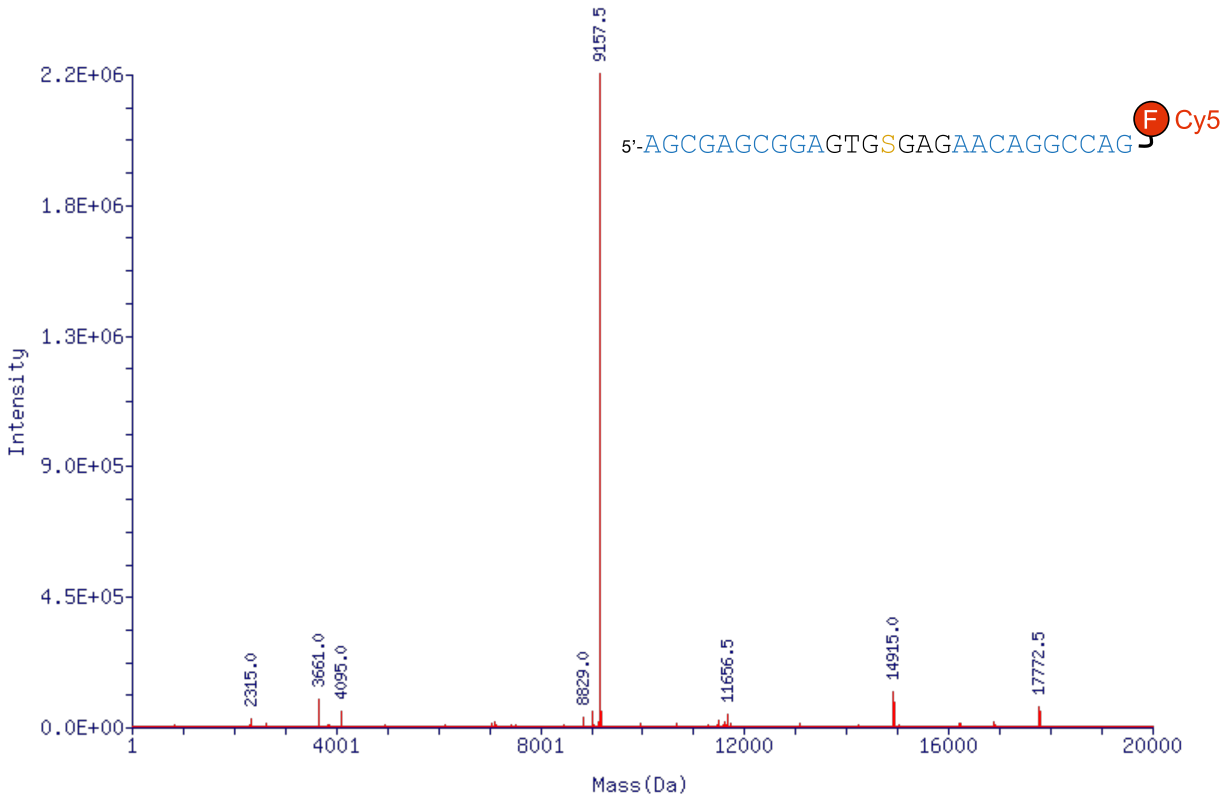

Figure S17. ESI-MS of AP-4 (top strand). S = THF modification. Mass calculated: 9157.8 Da; Mass found: 9157.5 Da.

Figure S18

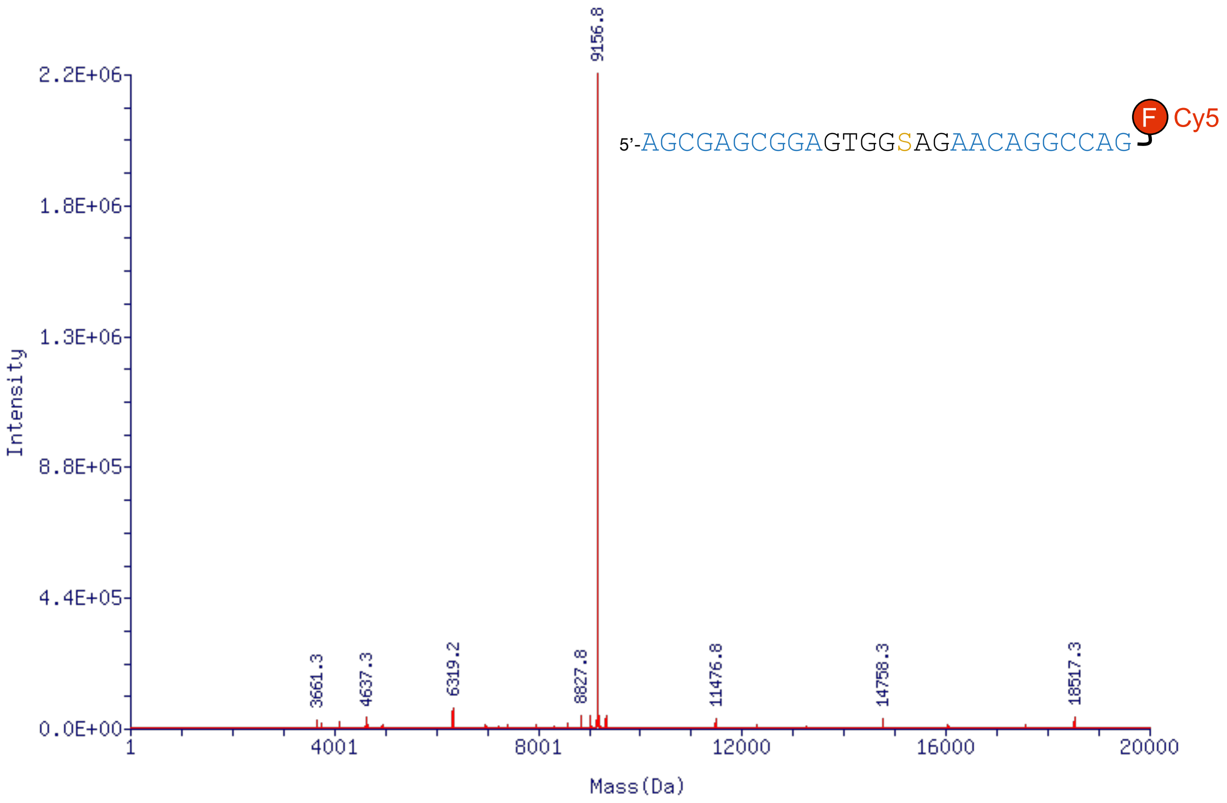

Figure S18. ESI-MS of AP-5 (top strand). S = THF modification. Mass calculated: 9157.8 Da; Mass found: 9156.8 Da.

Figure S19

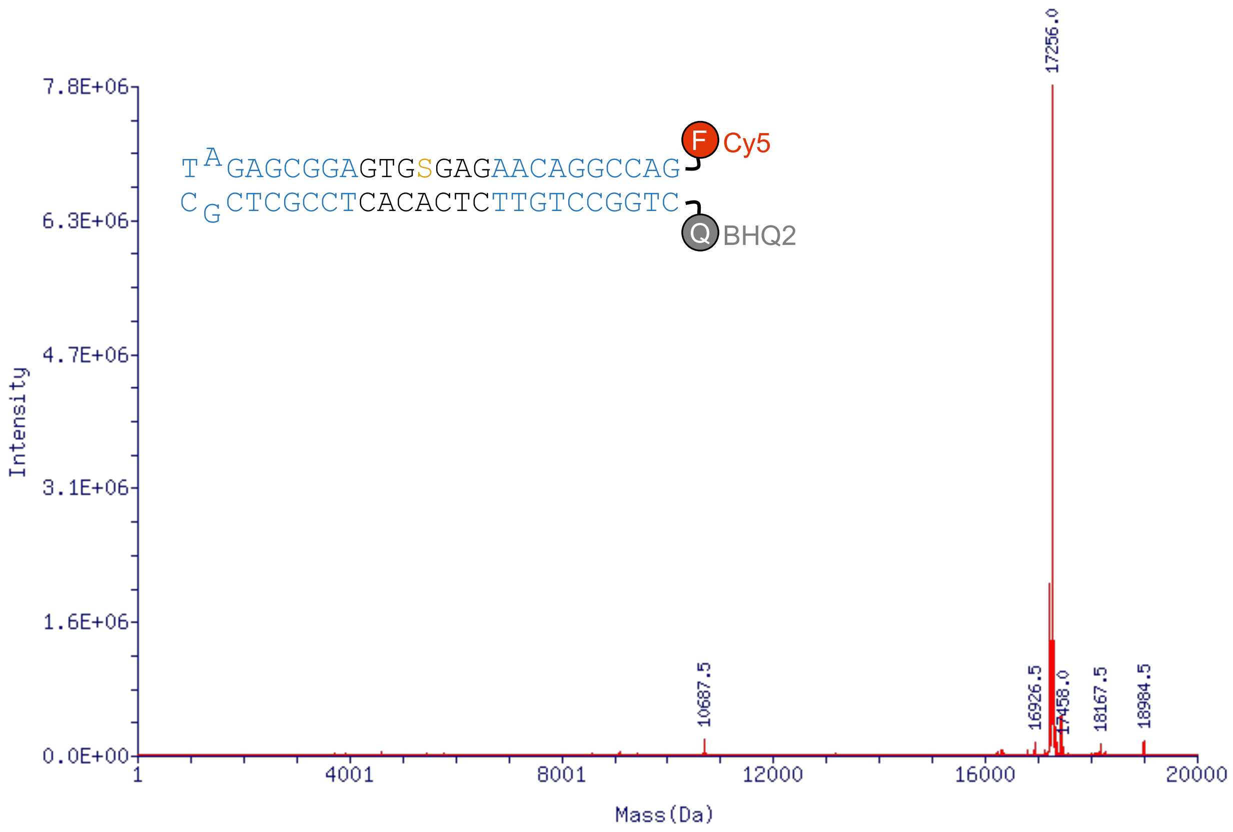

Figure S19. ESI-MS of AP-10. S = THF modification. Mass calculated: 17256.1 Da; Mass found: 17256.0 Da.

Figure S20

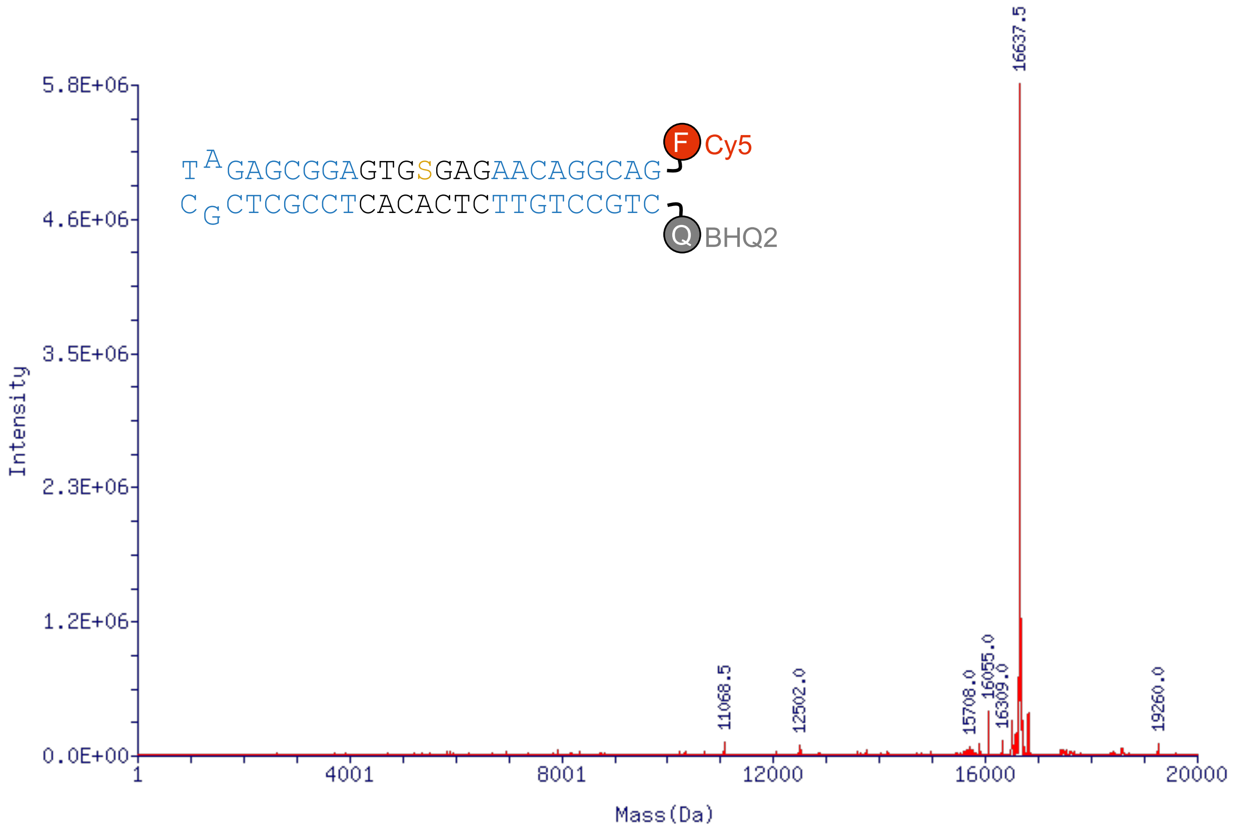

Figure S20. ESI-MS of AP-9. S = THF modification. Mass calculated: 16637.7 Da; Mass found: 16637.5 Da.

Figure S21

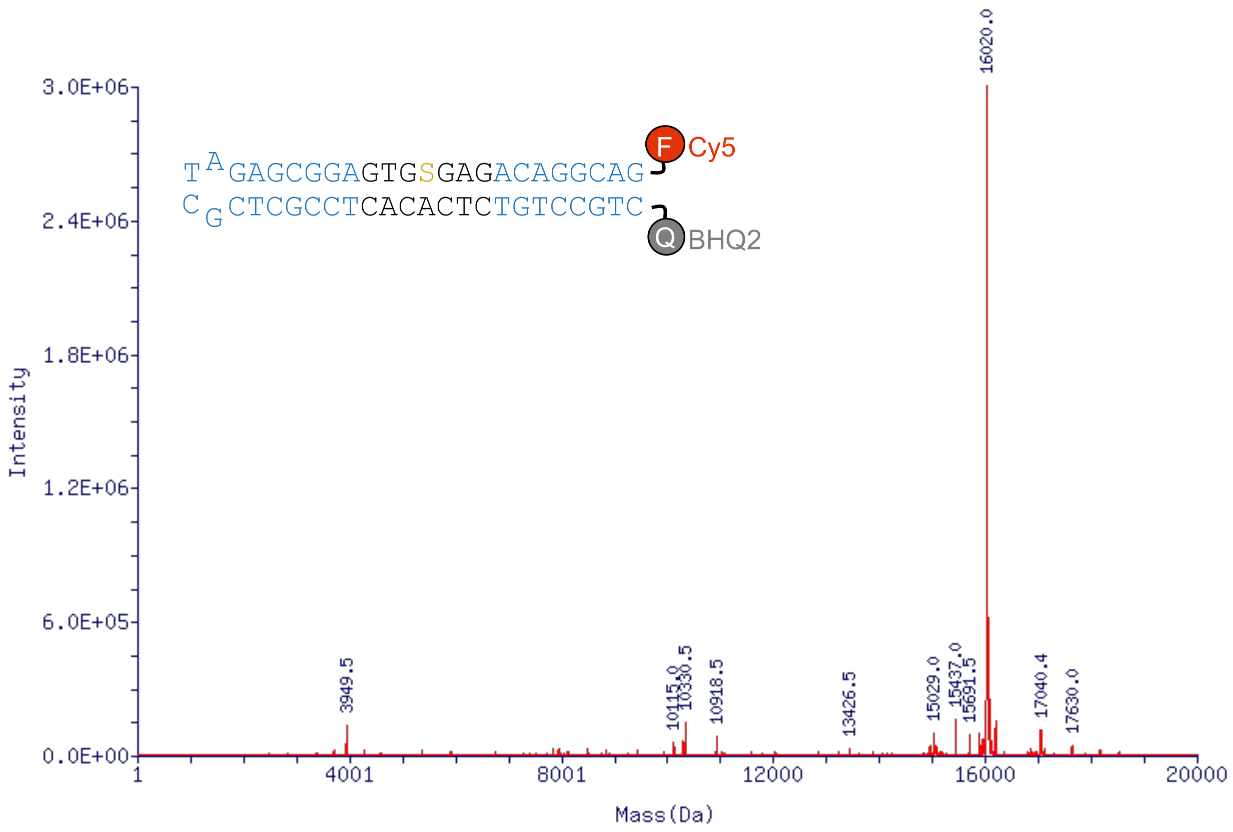

Figure S21. ESI-MS of AP-8. S = THF modification. Mass calculated: 16020.3 Da; Mass found: 16020.0 Da.

Figure S22

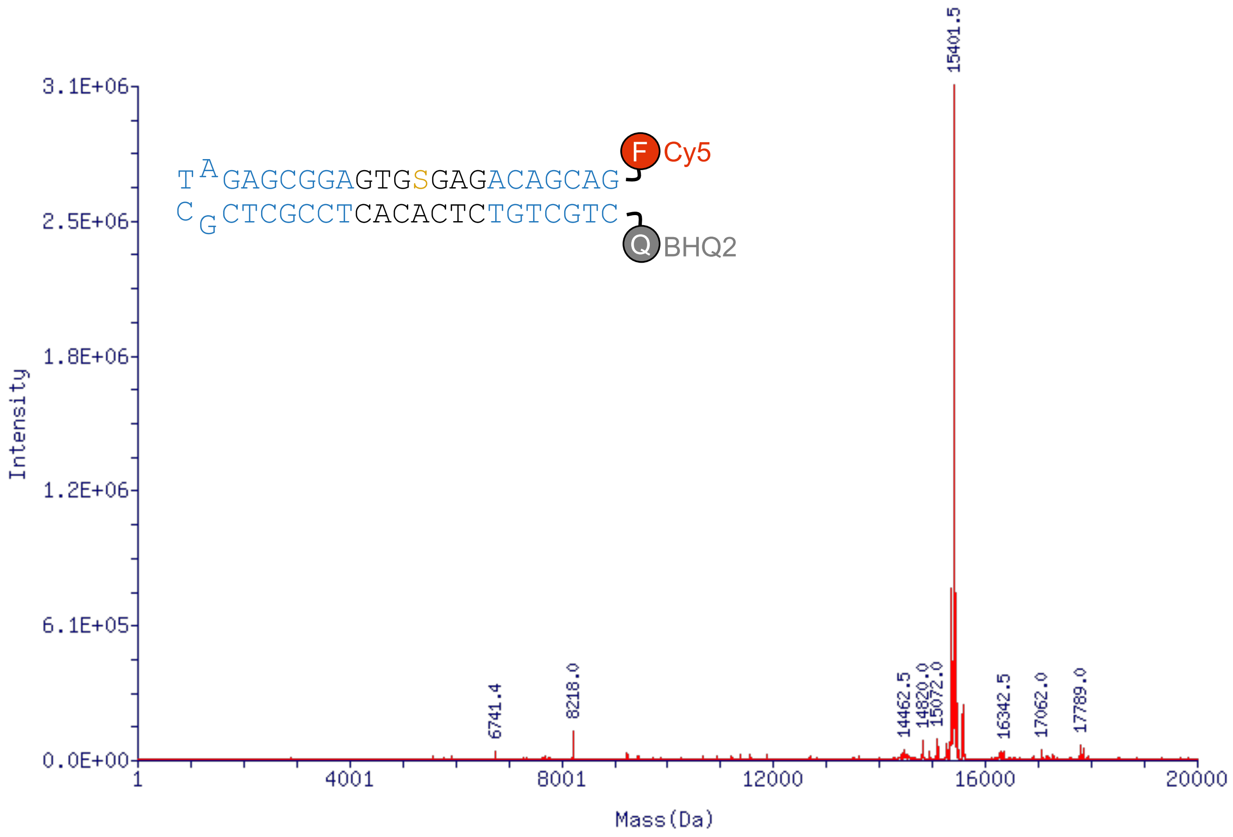

Figure S22. ESI-MS of AP-7. S = THF modification. Mass calculated: 15401.9 Da; Mass found: 15401.5 Da.

Figure S23

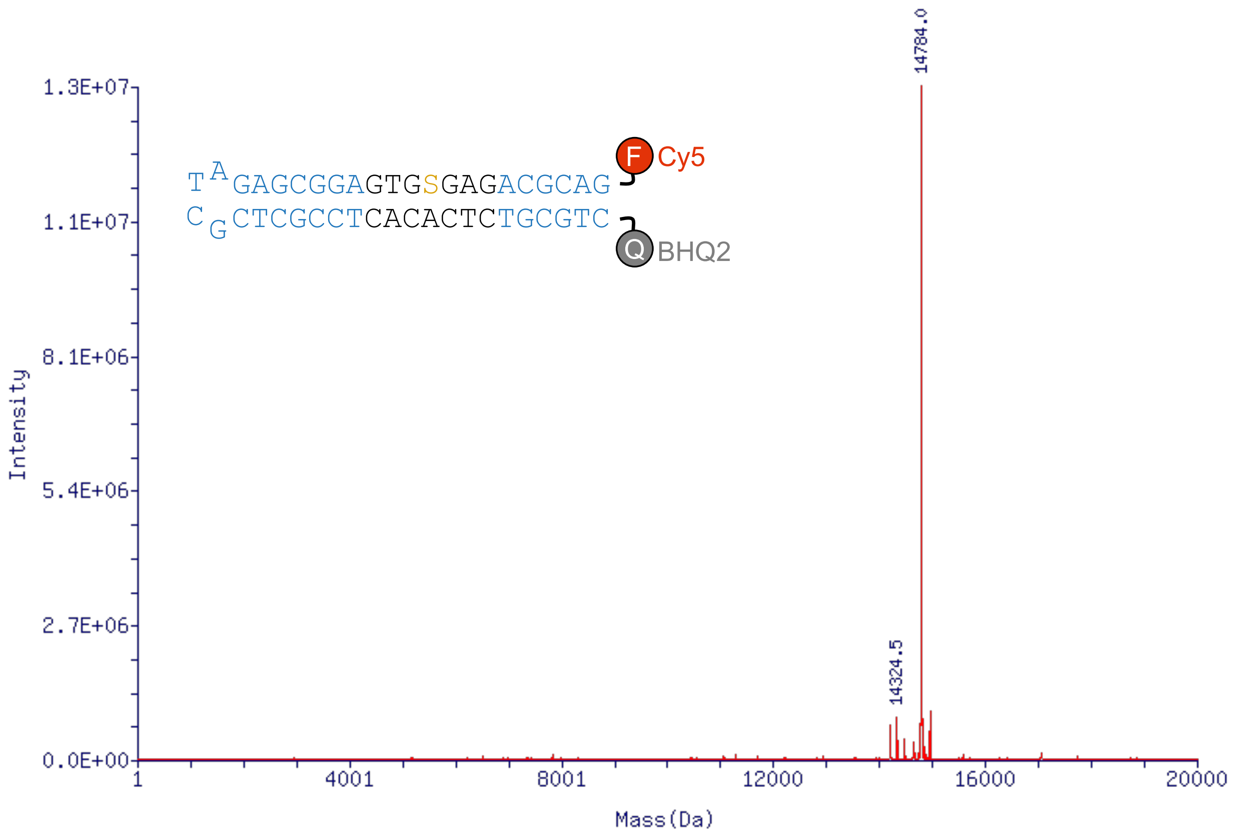

Figure S23. ESI-MS of AP-6. S = THF modification. Mass calculated: 14784.5 Da; Mass found: 14784.0 Da.

Figure S24

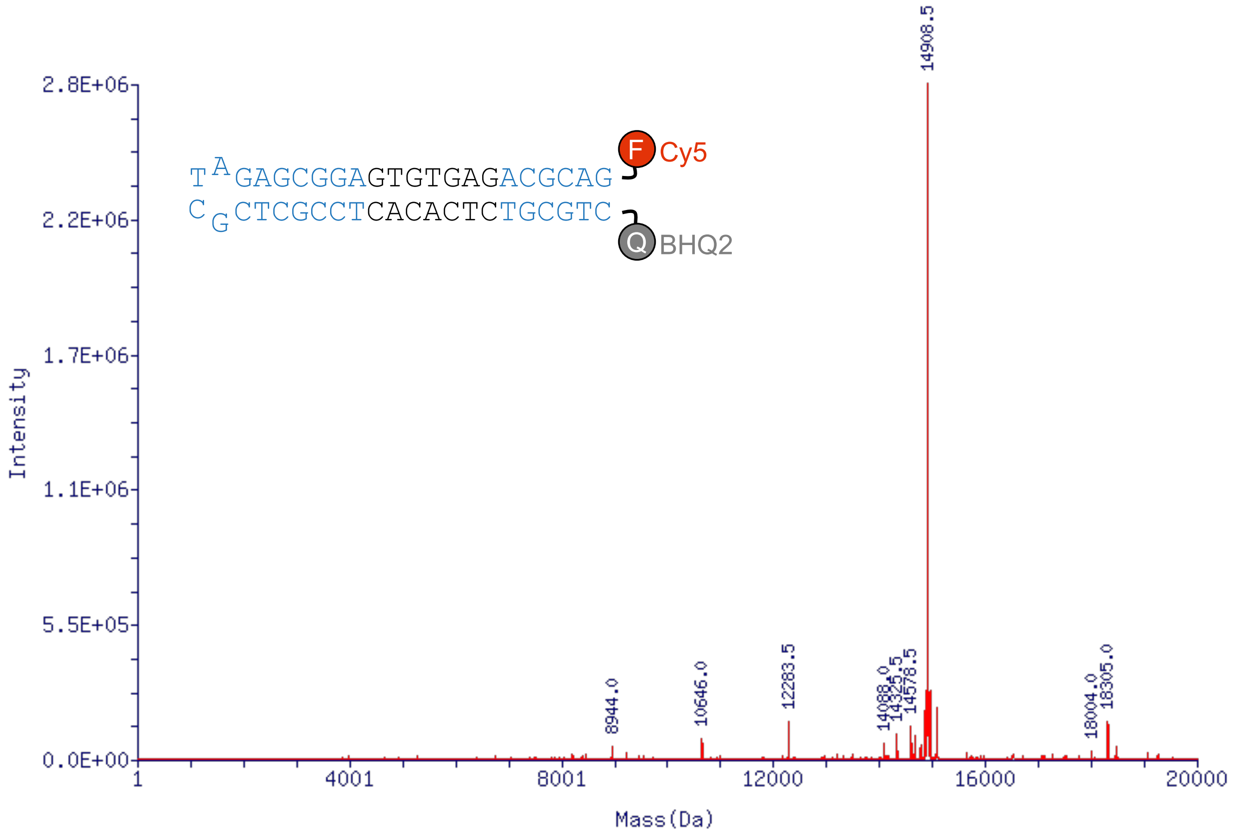

Figure S24. ESI-MS of T-6. Mass calculated: 14908.6 Da; Mass found: 14908.5 Da.

Figure S25

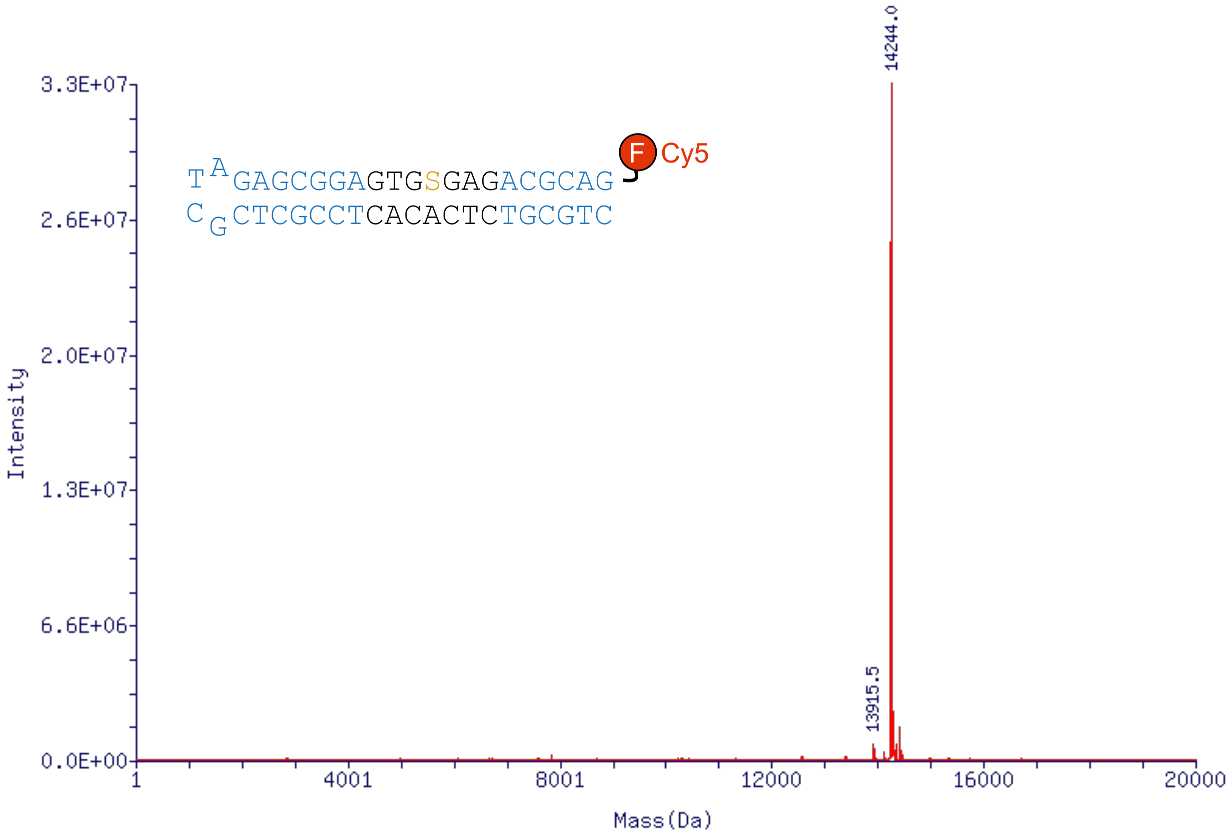

Figure S25. ESI-MS of AP-6_{NoQ}. S = THF modification. Mass calculated: 14244.1 Da; Mass found: 14244.0 Da.

Figure S26

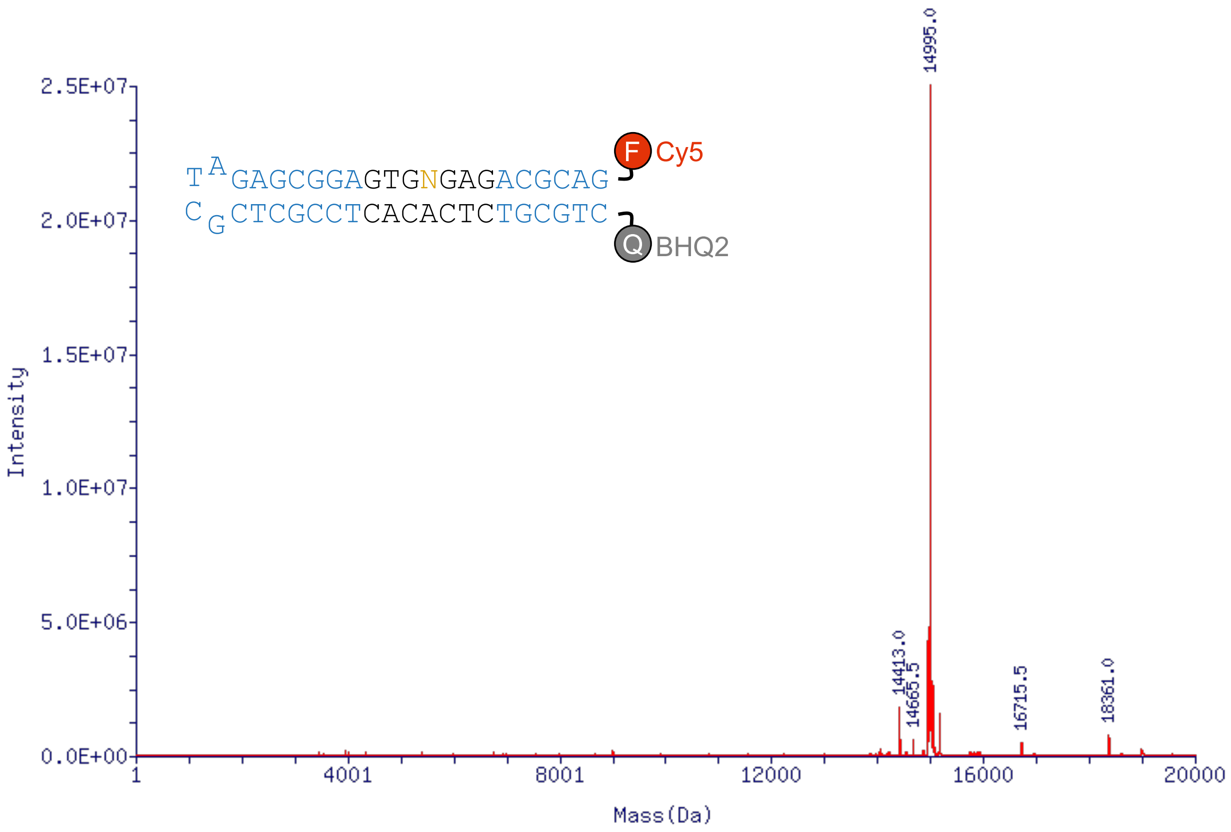

Figure S26. ESI-MS of N-6. N = AP_{NOV}. Mass calculated: 14994.5 Da; Mass found: 14995.0 Da.

Figure S27

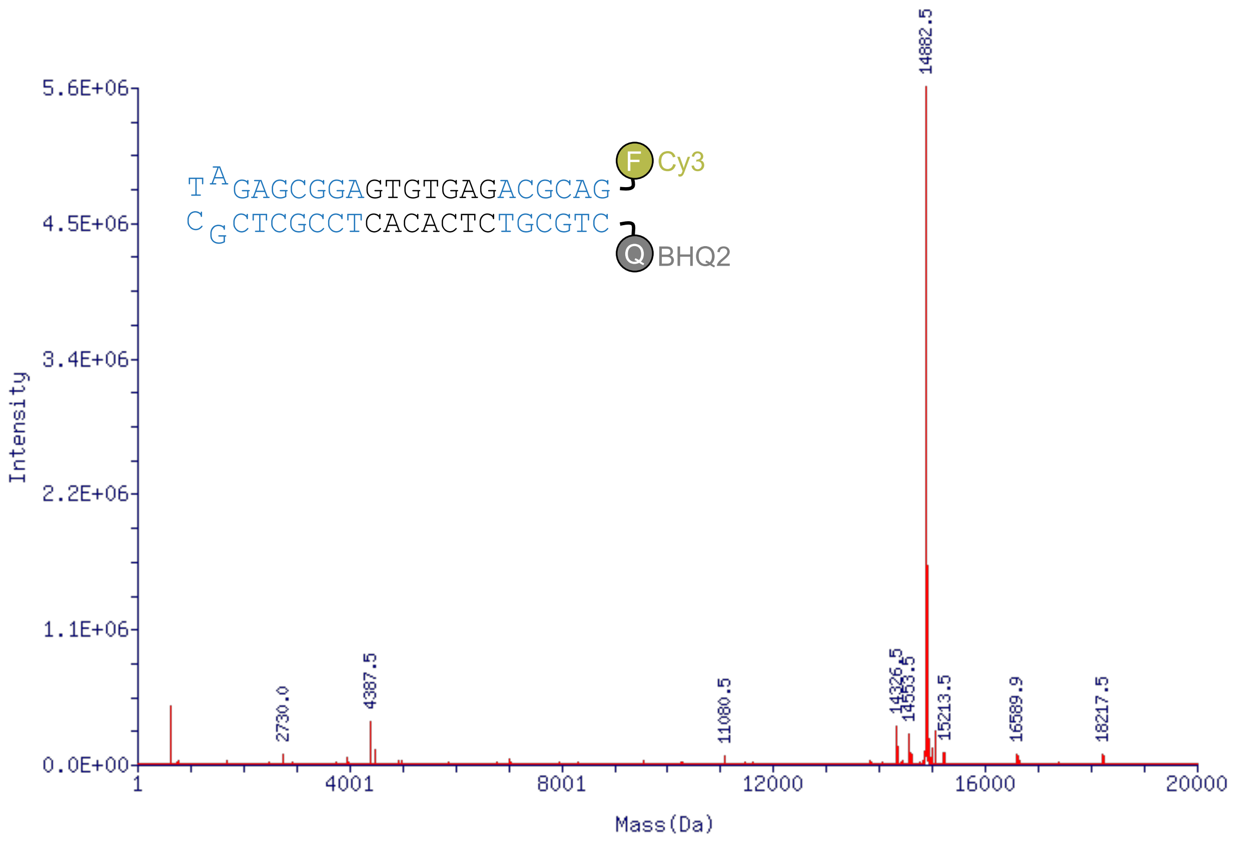

Figure S27. ESI-MS of T-6$_{Cy3}$. Mass calculated: 14882.6 Da; Mass found: 14882.5 Da.

Figure S28

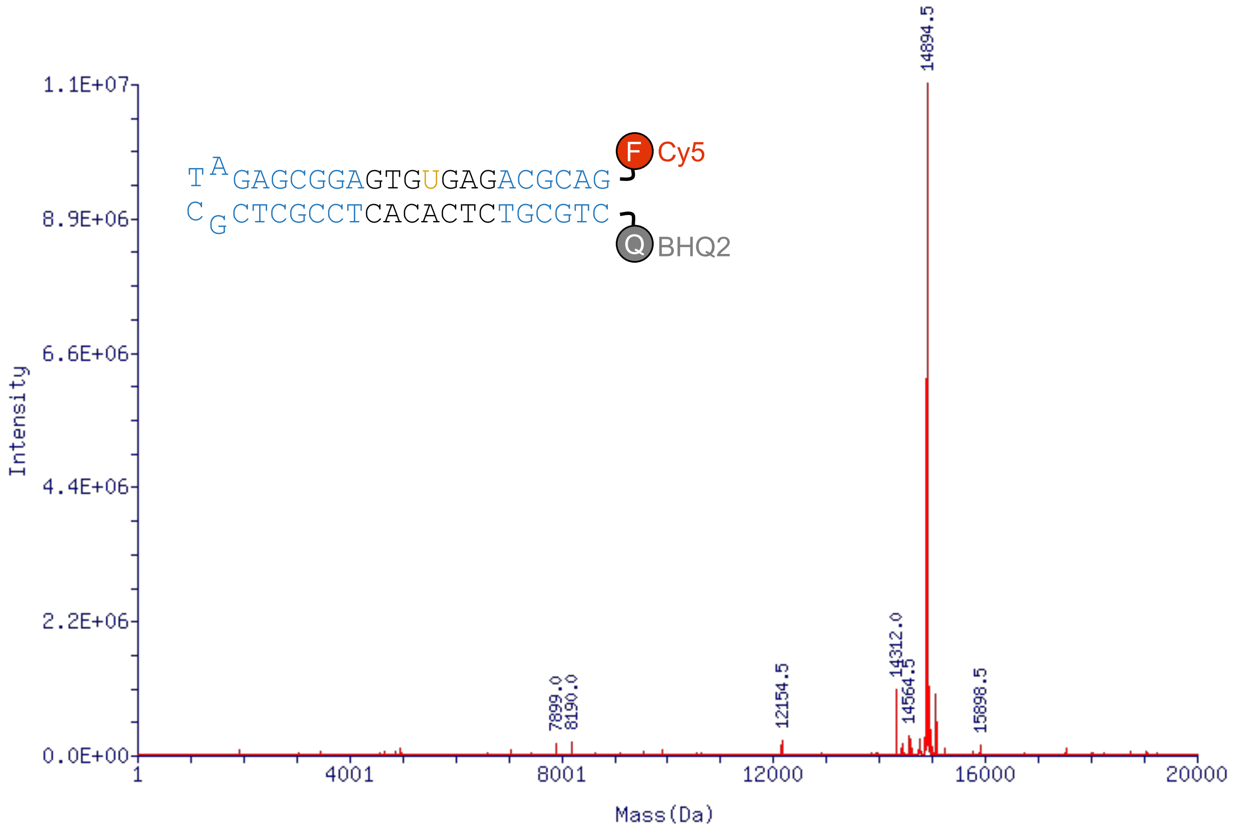

Figure S28. ESI-MS of U-6$_{Cy5}$. U = deoxyuridine. Mass calculated: 14894.5 Da; Mass found: 14894.5 Da.

Figure S29

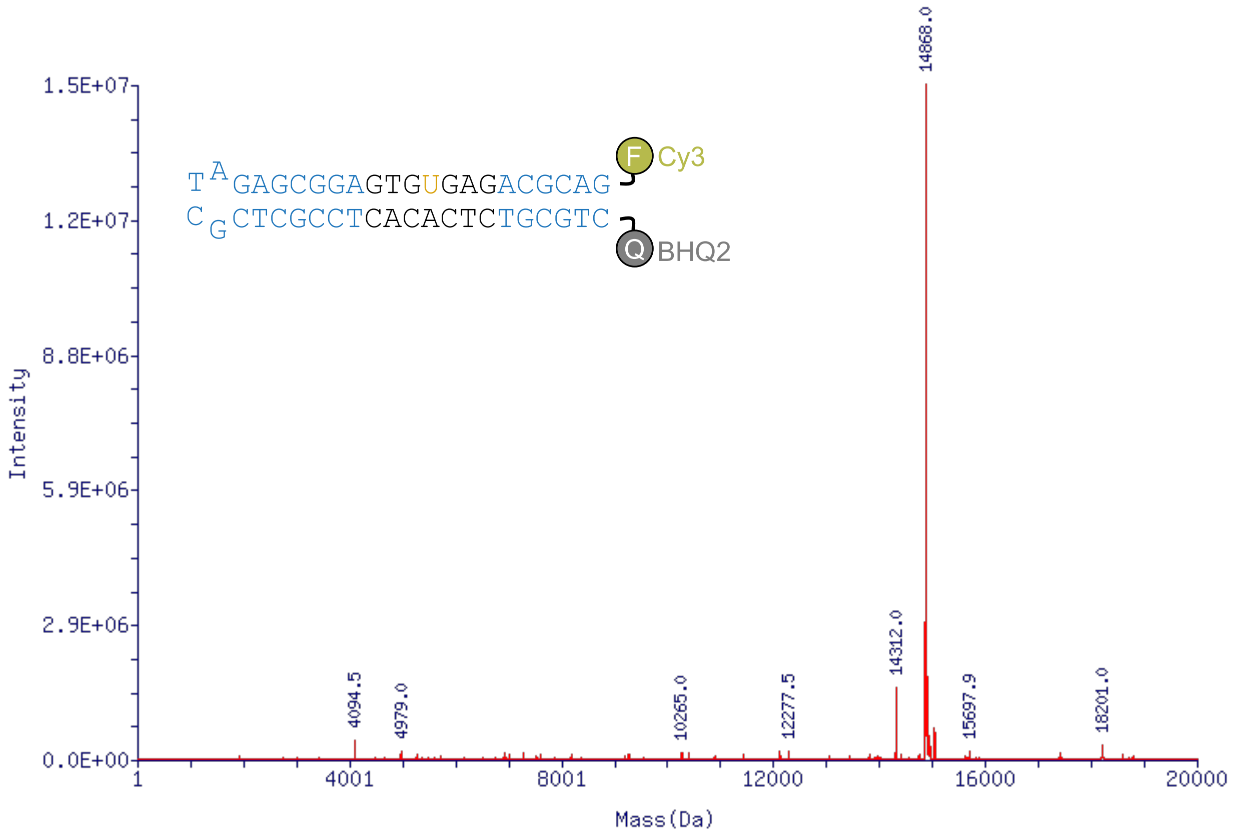

Figure S29. ESI-MS of U-6. U = deoxyuridine. Mass calculated: 14868.5 Da; Mass found: 14868.0 Da.

Figure S30

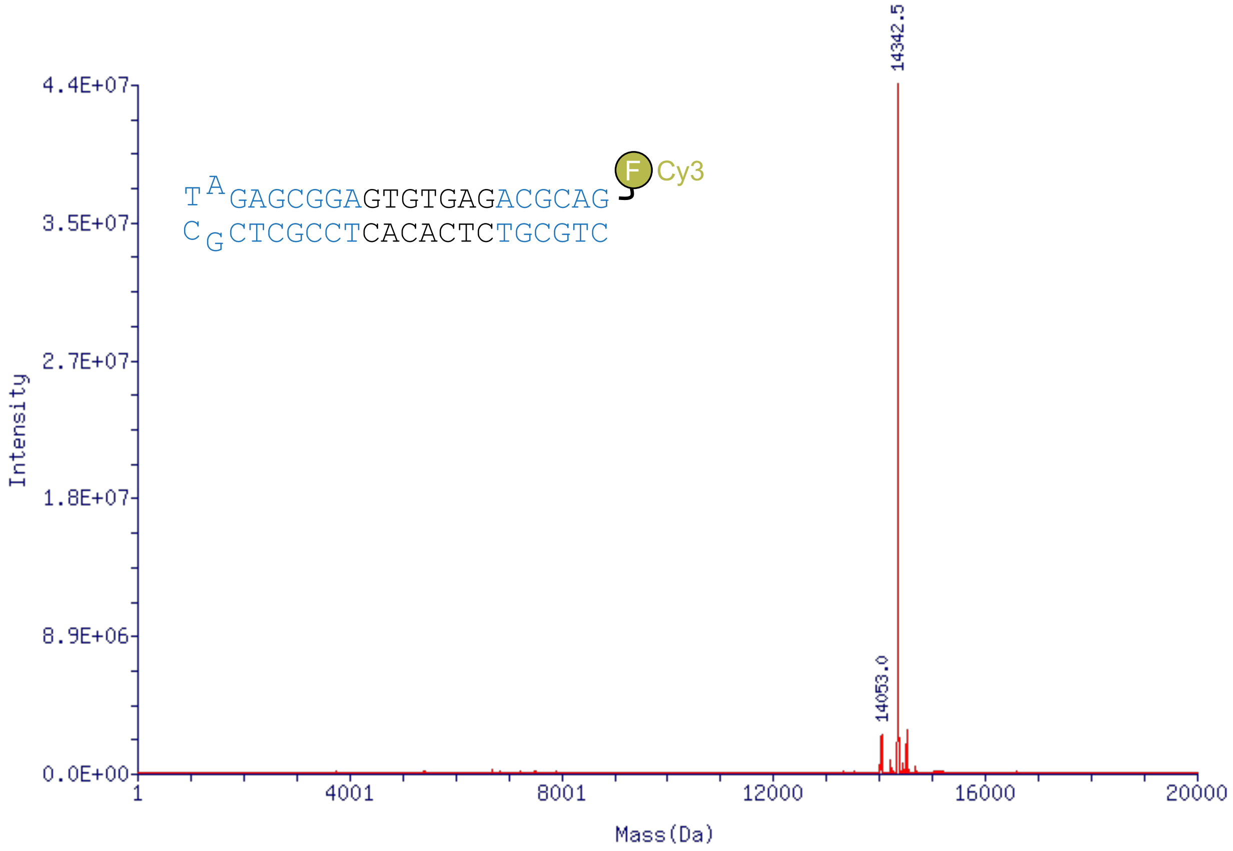

Figure S30. ESI-MS of T-$6_{Cy3\text{-}NoQ}$. Mass calculated: 14342.2 Da; Mass found: 14342.5 Da.

Figure S31

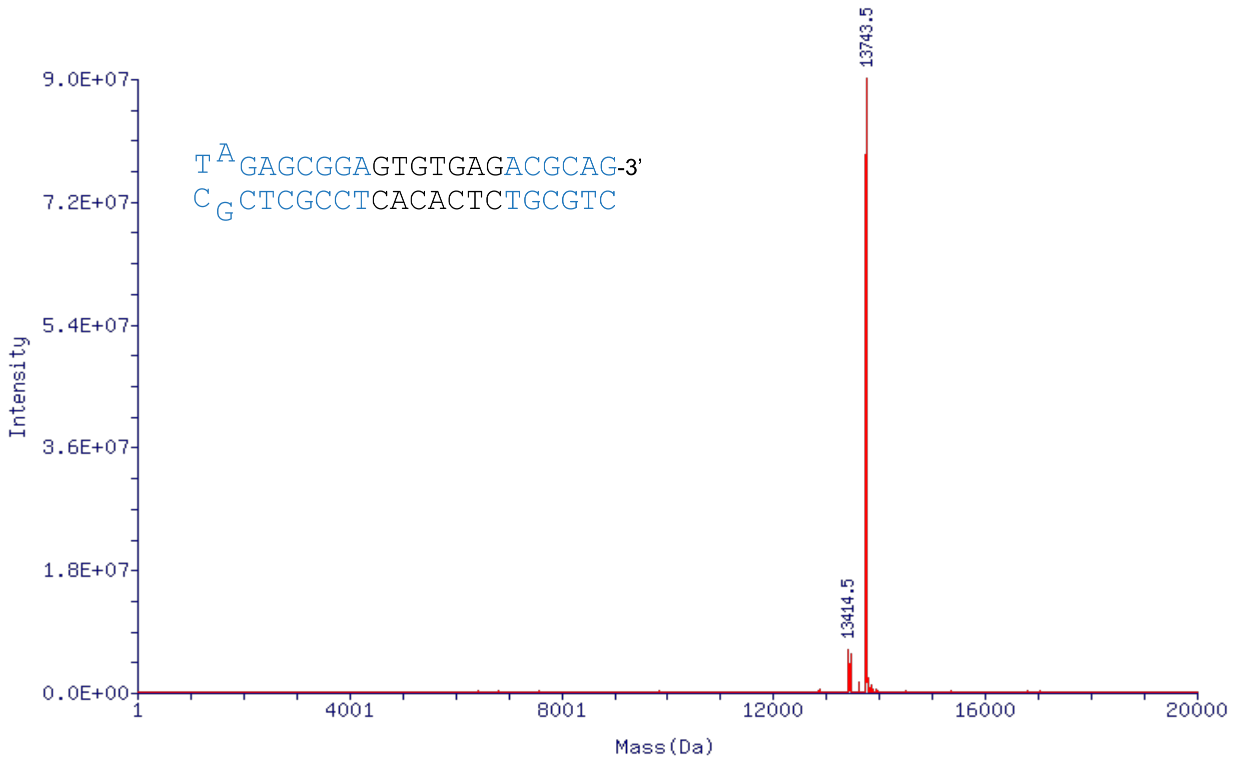

Figure S31. ESI-MS of T-$6_{NoDye\text{-}NoQ}$. Mass calculated: 13744.0 Da; Mass found: 13743.5 Da.

Figure S32

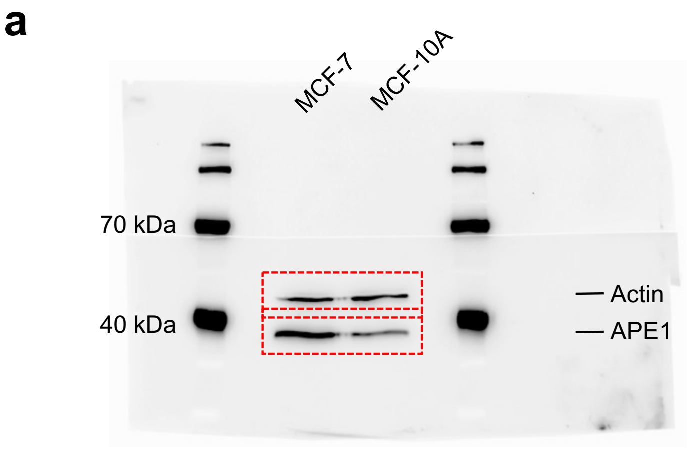

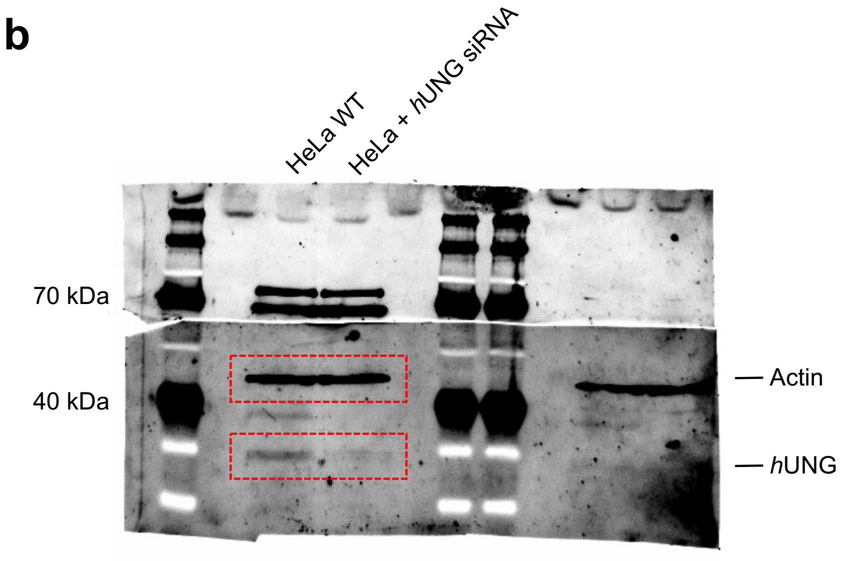

Figure S32. (a) Uncropped gel image for Figure S6b. Frames indicate the cropped regions shown in Figure S6b. (b) Uncropped gel image for Figure SDf. Frames indicate the cropped regions shown in Figure SDf.

S3. Supplementary Video

Caption for Supplementary Video File.

Probe N-6 was monitored using fluorescence microscopy by acquiring images just before UV irradiation (3 minutes), immediately after UV irradiation, and then every 2 minutes for 1 hour. Images (31 frames) were processed and chronologically stacked using ImageJ software (v1.54g). The resulting image stack was then exported as a time-lapse video at 5 frames per second. Scale bar = 25 μm.

S4. Supplementary Tables

Table S1. Names and sequences of all oligonucleotides used in this work. D-DNA (black) and L-DNA (blue) are indicated by color. S = THF; N = 4,5-dimethoxy-2-nitrobenzyl group; U = deoxyuridine; /Cy5/ = sulfo-Cyanine 5 dye; /Cy3/ = sulfo-Cyanine 3 dye; /BHQ2/ = Black Hole Quencher 2.

Sequence Name	Sequence Identity
D-AP-4	5'-AGCGAGCGGAGTGSGAGAACAGGCCAG/Cy5/<br>3'-TCGCTCGCCTCACACTCTTGTCCGGTC
AP-3	5'-AGCGAGCGGAGTSGGAGAACAGGCCAG/Cy5/<br>3'-TCGCTCGCCTCACACTCTTGTCCGGTC
AP-4	5'-AGCGAGCGGAGTGSGAGAACAGGCCAG/Cy5/<br>3'-TCGCTCGCCTCACACTCTTGTCCGGTC
AP-4$_{Q}$	5'-AGCGAGCGGAGTGSGAGAACAGGCCAG/Cy5/<br>3'-TCGCTCGCCTCACACTCTTGTCCGGTC/BHQ2/
AP-5	5'-AGCGAGCGGAGTGGSAGAACAGGCCAG-/Cy5/<br>3'-TCGCTCGCCTCACACTCTTGTCCGGTC
AP-10	5'-/BHQ2/CTGGCCTGTTCTCACACTCCGCTCGCTAGAGCGGAGTGSGAG AACAGGCCAG/Cy5/
AP-9	5'-/BHQ2/CTGCCTGTTCTCACACTCCGCTCGCTAGAGCGGAGTGSGAG AACAGGCAG/Cy5/
AP-8	5'-/BHQ2/CTGCCTGTCTCACACTCCGCTCGCTAGAGCGGAGTGSGAG ACAGGCAG/Cy5/
AP-7	5'-/BHQ2/CTGCTGTCTCACACTCCGCTCGCTAGAGCGGAGTGSGAG ACAGCAG/Cy5/
AP-6	5'-/BHQ2/CTGCGTCTCACACTCCGCTCGCTAGAGCGGAGTGSGAG ACGCAG/Cy5/
T-6	5'-/BHQ2/CTGCGTCTCACACTCCGCTCGCTAGAGCGGAGTGTGAG ACGCAG/Cy5/
AP-6$_{NoQ}$	5'-CTGCGTCTCACACTCCGCTCGCTAGAGCGGAGTGSGAG ACGCAG/Cy5/
N-6	5'-/BHQ2/CTGCGTCTCACACTCCGCTCGCTAGAGCGGAGTGNGAG ACGCAG/Cy5/
U-6	5'-/BHQ2/CTGCGTCTCACACTCCGCTCGCTAGAGCGGAGTGUGAG ACGCAG/Cy3/
T-6$_{Cy3}$	5'-/BHQ2/CTGCGTCTCACACTCCGCTCGCTAGAGCGGAGTGTGAG ACGCAG/Cy3/
T-6$_{Cy3\text{-}NoQ}$	5'-CTGCGTCTCACACTCCGCTCGCTAGAGCGGAGTGTGAG ACGCAG/Cy3/
T-6$_{NoDye\text{-}NoQ}$	5'-CTGCGTCTCACACTCCGCTCGCTAGAGCGGAGTGTGAG ACGCAG
U-6$_{Cy5}$	5'-/BHQ2/CTGCGTCTCACACTCCGCTCGCTAGAGCGGAGTGUGAG ACGCAG/Cy5/

S5. Supplementary Discussion

Creation of a chimeric beacon for monitoring *h*UNG activity. To generate a chimeric beacon for *h*UNG, we replaced the THF modification in AP-6 with a deoxyuridine (U) residue (Figure SDa and Table S1). This probe, U-6, first requires base excision of uracil by *h*UNG followed by APE1-mediated cleavage of the resulting AP site for activation. U-6 employed an A•U pair so that it would be identical to AP-6 except for the identity of the DNA lesion. No further sequence optimization was attempted. For the experiments described in the Supplementary Discussion, the probe was labeled with Cy5 (U-6_{Cy5}), whereas the probe used for the multiplexed assay (Figure 6) was labeled with Cy3 (U-6). We first verified that probe U-6_{Cy5} performed as intended in vitro using *E. coli* UDG (*e*UDG), which shares high sequence and structural similarities with *h*UNG (Figure SDb,c).[5-7] We then assessed the ability of U-6_{Cy5} to monitor *h*UNG activity in living HeLa cells. Compared to the undamaged T-6 control, transfection of the U containing probe U-6_{Cy5} resulted in bright fluorescence signal (Figure SDd), indicating *h*UNG-induced fluorescence activation. Additionally, fluorescence activation of U-6_{Cy5} was reduced to near background levels upon siRNA-mediated knockdown of *h*UNG, consistent with *h*UNG being the major repair enzyme for excision of U from DNA (Figure SDe,f).[8] These data were further confirmed by flow cytometry analysis (Figure SDg) and by gel electrophoresis of the extracted probes (Figure SDh). Taken together, these results confirm that probe U-6_{Cy5} can selectively monitor *h*UNG activity in living cells and highlight the adaptability of the chimeric beacon probe for other BER enzymes.

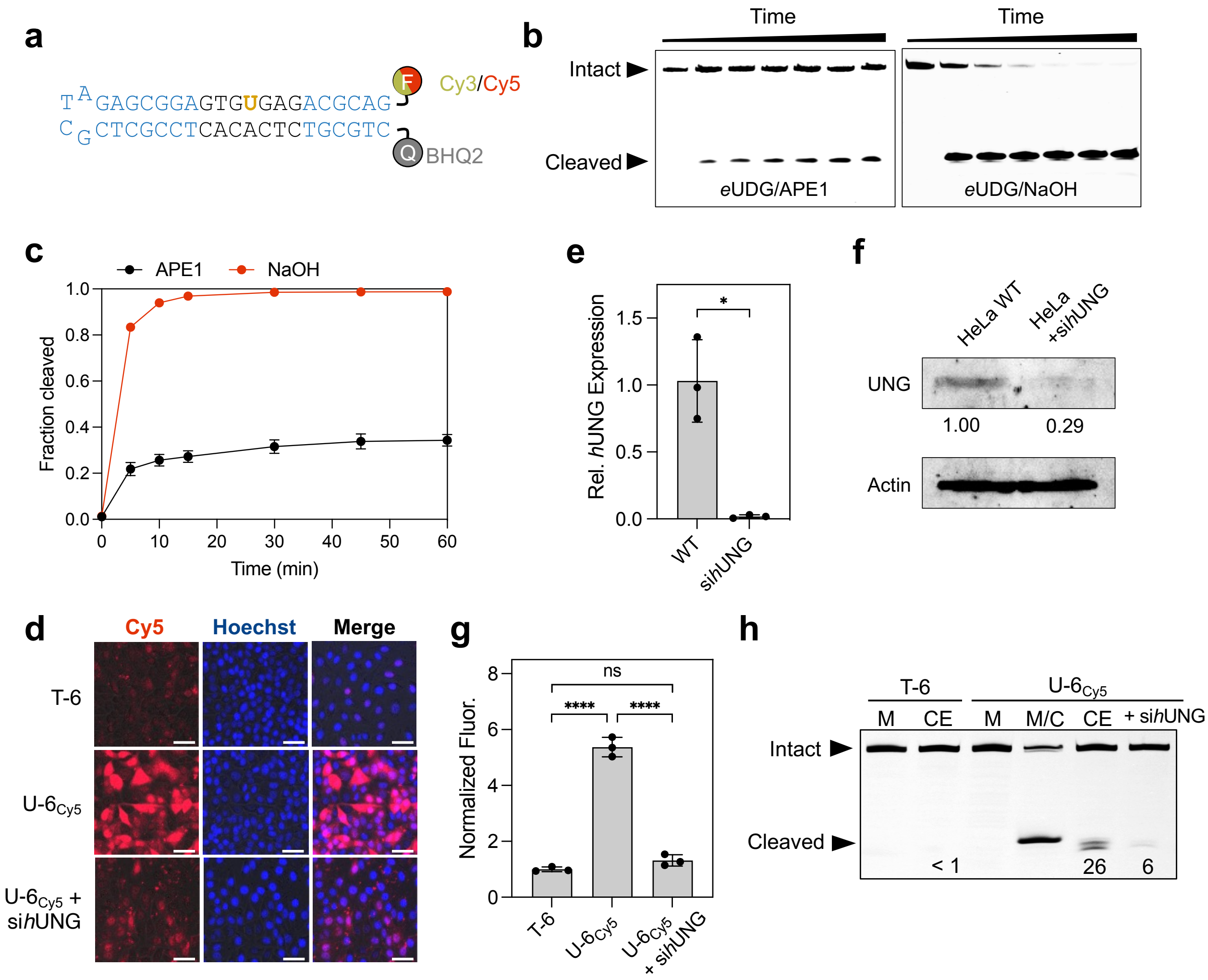

Figure SD. (a) A chimeric hairpin probe for *h*UNG. (b) Kinetic time course of probe U-6$_{Cy5}$ (200 nM) treated with 0.4 nM *e*UDG alone or together with 1 nM APE1 in a reaction buffer containing Tris-HCl (pH 8.0), 0.5 mM $Mg(CH_3COO)_2$, 1 mM EDTA, and 1 mM DTT at 37 °C. The AP sites in *e*UDG alone reactions were cleaved by heating at 90 °C in the presence of 0.1 M NaOH. (c) Plotting of the data represented in panel a. Error bars shows standard deviation (n = 3). (d) HeLa cells were transfected with 10 pmol of either T-6 or U-6$_{Cy5}$ and imaged by fluorescence microscopy 6 hours later. (+) si*h*UNG indicates cells that were treated with *h*UNG siRNA (both isoforms) 2 days prior to probe transfection. Scale bar = 50 µm. (e) RT-qPCR verification of *h*UNG knockdown by siRNA. Relative expression was calculated using the $2^{-\Delta\Delta Ct}$ method and normalized to *h*UNG in untreated cells. Error bars show standard deviation (n = 3 biological replicates). **P* < 0.05. (f) Western blot analysis of hUNG protein level in HeLa cells following siRNA knockdown. Values below each lane indicate the relative *h*UNG protein levels (normalized to actin). Uncropped gel

images are presented in Figure S32b. (g) Cells were treated under the same conditions described in panel b and analyzed by flow cytometry. Data is normalized to the T-6 control. Error bar shows standard deviation (n = 3 biological replicates). ****P < 0.0001. (h) Denaturing PAGE analysis of probes extracted from HeLa cells. M = marker for the intact probe; M/C = marker for the cleaved probe; CE = cell extract; (+) si*h*UNG = extract from *h*UNG siRNA treated cells. Values below CE lanes indicate the percent of cleaved probe.

S6. Supplementary References

1. Sczepanski, J. T.; Wong, R. S.; McKnight, J. N.; Bowman, G. D.; Greenberg, M. M., Rapid DNA-protein cross-linking and strand scission by an abasic site in a nucleosome core particle. *Proc Natl Acad Sci U S A* **2010,** *107* (52), 22475-80.
2. Yu, C.-H.; Sczepanski, J. T., The influence of chirality on the behavior of oligonucleotides inside cells: revealing the potent cytotoxicity of G-rich l-RNA. *Chemical Science* **2023,** *14* (5), 1145-1154.
3. Zhong, W.; Sczepanski, J. T., Chimeric d/l-DNA Probes of Base Excision Repair Enable Real-Time Monitoring of Thymine DNA Glycosylase Activity in Live Cells. *Journal of the American Chemical Society* **2023,** *145* (31), 17066-17074.
4. Mirbahai, L.; Kershaw, R. M.; Green, R. M.; Hayden, R. E.; Meldrum, R. A.; Hodges, N. J., Use of a molecular beacon to track the activity of base excision repair protein OGG1 in live cells. *DNA Repair* **2010,** *9* (2), 144-152.
5. Olsen, L. C.; Aasland, R.; Wittwer, C. U.; Krokan, H. E.; Helland, D. E., Molecular cloning of human uracil-DNA glycosylase, a highly conserved DNA repair enzyme. *The EMBO Journal* **1989,** *8* (10), 3121-3125.
6. Xiao, G.; Tordova, M.; Jagadeesh, J.; Drohat, A. C.; Stivers, J. T.; Gilliland, G. L., Crystal structure of Escherichia coli uracil DNA glycosylase and its complexes with uracil and glycerol: Structure and glycosylase mechanism revisited. *Proteins: Structure, Function, and Bioinformatics* **1999,** *35* (1), 13-24.
7. Ono, T.; Edwards, S. K.; Wang, S.; Jiang, W.; Kool, E. T., Monitoring eukaryotic and bacterial UDG repair activity with DNA-multifluorophore sensors. *Nucleic Acids Research* **2013,** *41* (12), e127-e127.
8. Kavli, B.; Sundheim, O.; Akbari, M.; Otterlei, M.; Nilsen, H.; Skorpen, F.; Aas, P. A.; Hagen, L.; Krokan, H. E.; Slupphaug, G., hUNG2 Is the Major Repair Enzyme for Removal of Uracil from U:A Matches, U:G Mismatches, and U in Single-stranded DNA, with hSMUG1 as a Broad Specificity Backup. *Journal of Biological Chemistry* **2002,** *277* (42), 39926-39936.
